# Discordant Chloroplast and Nuclear Divergence Among Eight *Rubus crataegifolius* (Rosaceae) Cultivars

**DOI:** 10.3390/genes17101287

**Published:** 2026-10-15

**Authors:** Seol-Jong Kim, Yoon-Young Kim, Soon-Ho Kwon

**Affiliations:** National Forest Seed Variety Center (NFSV), Chungju 27495, Republic of Korea; seoljong.kim@gmail.com (S.-J.K.); epyoony@gmail.com (Y.-Y.K.)

**Keywords:** cultivar identification, cytonuclear discordance, maternal lineage, nuclear SNP, plastome, *Rubus crataegifolius*

## Abstract

**Background**: Cultivar identification and the reconstruction of breeding relationships require markers that reflect both parents, yet plastome sequencing is often applied to these problems on its own. **Methods**: We tested how far chloroplast data alone can go in *Rubus crataegifolius* Bunge, using one plant of each of eight cultivars from Korean breeding programs. Complete chloroplast genomes (155,664–155,770 bp) were assembled for all eight and compared with nuclear resequencing data from the same plants. **Results**: The 526 plastome variants were concentrated in non-coding regions, and Ka/Ks ratios well below one indicated strong purifying selection on plastid genes. The eight cultivars carried only five distinct plastome haplotypes: three shared one identical sequence and two another. Mapping to a *Rubus idaeus* reference recovered 4,872,213 cultivar-polymorphic nuclear SNPs, which separated every cultivar pair, including all four with identical plastomes, and the result was unchanged after stringent filtering of heterozygous calls. Cultivars sharing an identical plastome spanned 27% of the entire range of nuclear distances, and the two measures were only weakly correlated. **Conclusions**: Plastome sequencing therefore describes maternal lineage rather than cultivar identity among these cultivars; genome-wide nuclear SNPs discriminate them, and the five-SNP panel derived from them is a candidate tool that requires validation on independent plants.

## 1. Introduction

The genus *Rubus* L. (Rosaceae) is one of the most taxonomically complex genera of flowering plants, comprising approximately 700–1000 species distributed worldwide [1,2]. The classification of *Rubus* has long been challenging due to extensive hybridization, polyploidy, apomixis, and morphological variability [1]. *Rubus crataegifolius* Bunge, commonly known as Korean raspberry, is native to East Asia and distributed across Korea, China, Japan, and the Russian Far East [3]. As a representative species of the ecologically and taxonomically significant genus *Rubus* in the Asia–Pacific region, *R. crataegifolius* is ecologically important as a wild fruit resource and has been the subject of active cultivar development in Korea. Nevertheless, the genomic relationships among its cultivars remain largely uncharacterized. Distinguishing these cultivars and reconstructing how they are related to one another is a practical requirement for variety protection and for the management of breeding material.

Chloroplast (cp) genomes have emerged as powerful tools for plant systematics and evolutionary studies due to their conserved structure, uniparental inheritance, and moderate evolutionary rates [4]. Thousands of plastomes are now available, and comparative analyses of them have clarified both the dynamics of plastome evolution and the limits of the plastid signal, including its frequent discordance with nuclear data [5,6]. At the deepest level, plastid phylogenomics has produced a family-level framework for the flowering plants [7]; at the shallowest, dense sampling of plastomes within a single crop—the pan-plastome—now resolves domestication history, geographic structure, and hypervariable barcoding regions [8], and plastome phylogenomics has been used to identify the maternal progenitor of cultivated chrysanthemum [9]. Complete cp genome sequences have thus been widely used to resolve phylogenetic relationships at various taxonomic levels, including intraspecific variation [10]. In *Rubus*, several cp genomes have been reported, including *R. crataegifolius* [11], *Rubus takesimensis*, an anagenetically derived species endemic to Ulleung Island, Korea [12], and comparative studies across the genus [13,14,15]. However, intraspecific cp genome variation within *R. crataegifolius* cultivars has not been investigated.

Because the plastome is maternally inherited in most angiosperms and effectively non-recombining, it records the seed parent alone (maternal inheritance has not been tested directly in *R. crataegifolius*, and the inference below assumes it). Cultivars derived from a common seed parent but different pollen parents are expected to carry indistinguishable plastomes despite differing nuclear backgrounds, and reciprocal crosses produce the opposite pattern. Plastome data therefore cannot, in principle, establish cultivar identity or overall genetic distance, although they are frequently used for these purposes. How severe this limitation is in practice can only be judged by comparing plastome and nuclear data from the same plants, which has rarely been done at the cultivar level in *Rubus*. Where such a comparison has been made in a berry crop, the two genomes told different stories: northern and southern highbush blueberries are close in nuclear terms yet carry plastomes of distinct maternal origin [16]. For cultivar identification in *Rubus*, a 48-assay KASP fingerprinting panel, reducible to a minimal set of 24 assays, is available for *Rubus idaeus* [17], but no comparable marker set exists for *R. crataegifolius*. For the same eight cultivars examined here, geometric morphometric analysis of leaf shape classified the cultivars with 72.0% mean accuracy (87.2% for landmark-based analysis of primocane leaves), which the authors of that study regarded as a preliminary screening tool rather than a substitute for molecular identification [18].

Here we assembled the complete cp genomes of eight *R. crataegifolius* cultivars and generated nuclear resequencing data for the same eight plants. Our objectives were to (1) characterize plastome structure, repeat content and sequence variation among the cultivars, (2) test whether plastome variation is sufficient to distinguish them, (3) quantify the agreement between plastome-based and nuclear genome-based estimates of cultivar relationships, and (4) assess whether nuclear SNPs provide sufficient resolution for marker-based cultivar identification. Seven of the eight cultivars were developed by one regional breeding program and the eighth by another, and none was sampled to represent the natural genetic diversity of the species; the results below therefore describe the relationships among these cultivars rather than the intraspecific genomic landscape of *R. crataegifolius* as a whole.

## 2. Materials and Methods

### 2.1. Plant Materials and DNA Extraction

Eight cultivars of *R. crataegifolius* Bunge maintained at the National Forest Seed Variety Center (NFSV), Chungju, Republic of Korea, were used in this study. One plant was sampled per cultivar. All eight cultivars are maintained at NFSV as clones propagated by root cuttings, so a single plant carries the cultivar genotype, as for other clonally propagated *Rubus* cultivars [17]; the analyses below therefore describe these eight cultivars as represented by one plant each, and variation among plants of the same cultivar, whether from somatic mutation or from mislabeled stock, was not assessed. Four are registered cultivars (‘Dongakheuk’, ‘Dongakhong’, ‘Geumdongcheon’, and ‘Geumdongwang’) and four have been filed for plant variety protection (‘Dabok’, ‘Daesung’, ‘Goldmoon’, and ‘Hwangeumboll’). All except ‘Goldmoon’ were developed by the Gyeongsangbuk-do Agricultural Research and Extension Services; ‘Goldmoon’ was developed by the Jeonnam Forest Research Institute [18]. Voucher specimens of all cultivars except ‘Hwangeumboll’ were deposited at NFSV (voucher numbers 0300-06-007949–0300-06-007955); no voucher was deposited for ‘Hwangeumboll’ because insufficient material was available at the time of collection. Total genomic DNA was extracted from fresh leaf tissue using the DNeasy Plant Mini Kit (Qiagen, Hilden, Germany). Whole-genome sequencing was performed on the NovaSeq 6000 platform (Illumina, San Diego, CA, USA), generating 151 bp paired-end reads. Between 19.9 and 23.8 million read pairs were obtained per cultivar (5.9–6.9 Gb after trimming; 51.9 Gb in total).

### 2.2. Chloroplast Genome Assembly and Annotation

Raw reads were quality-trimmed using Trimmomatic v0.40 [19] in paired-end mode with ILLUMINACLIP:TruSeq3-PE.fa:2:30:10, LEADING:3, TRAILING:3, SLIDINGWINDOW:4:15, and MINLEN:36; 96.0–98.1% of read pairs survived trimming. Complete cp genomes were assembled de novo using GetOrganelle v1.7.7.1 [20] with the previously published *R. crataegifolius* cp genome [11] as a reference seed. All eight assemblies yielded single circular molecules at an average plastid base coverage of 114–178×. The cp genomes were standardized by orienting the SSC region in the forward direction and rotating sequences to begin at the *trnH*-GUG gene, following convention.

Genome annotation was performed using GeSeq [21] (https://chlorobox.mpimp-golm.mpg.de/geseq.html, accessed on 4 March 2026) with manual curation. Gene boundaries were verified by comparison with the reference cp genome (NC_039704). The annotated sequences have been deposited in GenBank under accession numbers PZ143672–PZ143679.

### 2.3. Identification of Repeat Elements and Codon Usage Analysis

Simple sequence repeats (SSRs) were identified using MISA v2.1 [22,23] with the following minimum repeat thresholds: mononucleotide-10, dinucleotide-5, trinucleotide-4, tetranucleotide-3, pentanucleotide-3, and hexanucleotide-3. Tandem repeats were detected using Tandem Repeats Finder v4.10 [24] with parameters 2 7 7 80 10 50 500. Dispersed repeats were identified by self-alignment using BLAST+ v2.17.0 [25] and classified into four types—forward (F), palindromic (P), reverse (R), and complement (C)—following the conventions of REPuter [26]. For each cp genome, BLASTn was run against itself with ungapped alignment, word size 7, e-value 1000, and both strands; the IR-derived palindromic repeat corresponding to the IRa/IRb pair was excluded from the counts; R and C repeats were detected by additional queries against reverse and complement copies of each genome, respectively. Hits of at least 30 bp with no more than three mismatches (Hamming distance ≤ 3) were retained, and large palindromic hits derived from the inverted repeat regions (>1 kb) were excluded from dispersed repeat counts. Inverted repeat boundaries were located from the GeSeq annotations by recording the position of the first and last annotated feature on either side of each junction. For codon usage analysis, relative synonymous codon usage (RSCU) values were calculated with a custom Python v3.12 script using Biopython v1.86 [27] for all protein-coding sequences (CDS) of each cultivar. To avoid redundancy from IR-duplicated genes, each CDS was counted only once. Stop codons were excluded from the analysis. To place the repeat content of the cultivars in context, the same three analyses were applied with identical parameters to the twelve published *Rubus* plastomes used in the phylogenetic analysis (Table S1). Length polymorphism of plastid SSR loci among the eight cultivars was assessed by mapping every MISA-called locus through the MAFFT alignment described below and recording its ungapped length in each cultivar, in NC_039704 and in *R. takesimensis* NC_037991.

### 2.4. Sequence Alignment and Variant Detection

The eight cp genome sequences were aligned using MAFFT v7.525 [28]. Single nucleotide polymorphisms (SNPs) and insertions/deletions (InDels) were identified from the alignment using a custom Python script. Variants were classified by genomic region (LSC, SSC, IRa, IRb) and by genic/intergenic location. Pairwise SNP distances were calculated for all cultivar pairs.

### 2.5. Selection Pressure Analysis

To test whether the observed distribution of variants reflects selection rather than differences in partition length, substitution rates were normalized by the length of each genomic partition (protein-coding sequences, tRNA/rRNA genes, and non-coding regions), as defined by the annotation of ‘Dongakhong’. Synonymous (Ks) and non-synonymous (Ka) substitution rates were estimated by the Nei–Gojobori method [29], as implemented in Biopython v1.86 [27]. The input was codon-aware alignments of the 79 protein-coding genes shared by all eight cultivars; codons containing gaps, ambiguous bases or stop codons were excluded pairwise. Estimates of Ka/Ks can be unstable at low divergence, so 95% confidence intervals for the concatenated values were obtained by resampling codon columns with replacement, using 1000 replicates for the interspecific comparisons and 300 for each cultivar pair. Ka/Ks was calculated per gene and for concatenated alignments, among the eight cultivars and between ‘Dongakhong’ and three congeners retrieved from GenBank (*R. takesimensis* NC_037991, *R. coreanus* MT478114, and *R. chingii* NC_050296).

### 2.6. Phylogenetic Analysis

For phylogenetic reconstruction, the eight *R. crataegifolius* cp genomes from this study were aligned with 12 published *Rubus* cp genomes from GenBank (Table S1) and one outgroup species (*Prunus mume*, MW759300) using MAFFT v7.525 [28], giving an alignment of 167,962 positions of which 12,648 were variable. A maximum likelihood (ML) tree was constructed using IQ-TREE v3.0.1 [30] with the TVM+F+R3 substitution model selected by ModelFinder under BIC [31]. Branch support was assessed with 1000 ultrafast bootstrap replicates [32] and 1000 SH-aLRT replicates, and is reported below as SH-aLRT/UFBoot. Because the plastome is a single non-recombining locus, multi-locus coalescent methods are not applicable to it; relationships among the plastome haplotypes were instead summarized as a minimum spanning network [33]. The eight cultivar plastomes, NC_039704, and *R. takesimensis* NC_037991 were taken from the same alignment, columns containing a gap or ambiguous base in any of the ten sequences were removed, identical sequences were collapsed into haplotypes, and the network was built from pairwise SNP counts with NetworkX v3.2.1 [34], retaining all minimum-spanning edges and any alternative edge no longer than the longest edge on the corresponding tree path.

### 2.7. Nuclear Resequencing and SNP Calling

To assess cultivar relationships from biparentally inherited markers, the quality-trimmed reads of all eight cultivars were mapped to the *R. idaeus* reference assembly drRubIdae1.hap1.1 (GenBank GCA_965153425.1; 274.8 Mb in 63 sequences) using bwa-mem2 v2.3 [35]. No chromosome-level assembly is available for *R. crataegifolius*. *R. idaeus* was chosen because it belongs to the same subgenus (*Idaeobatus*) and, in a pilot comparison of two candidate assemblies using two million read pairs of ‘Dongakheuk’, gave the higher mapping rate: 91.7%, against 86.9% for *R. idaeus* ‘Malling Jewel’ (GCA_030142095.1). Alignments were coordinate-sorted and duplicate-marked with SAMtools v1.24 [36], and duplicates were excluded from variant calling by the default read filters of BCFtools mpileup. Mean depth per cultivar was estimated as the number of primary mapped reads multiplied by the read length and divided by the reference length; it is therefore an approximation rather than a per-base measurement. Joint variant calling across all eight cultivars was performed with BCFtools v1.24 [36], using mpileup (minimum mapping quality 20, minimum base quality 20) followed by *call -mv*. The genome was divided into 5-Mb windows that were processed in parallel, and the resulting files were concatenated. Variants were retained if they were biallelic SNPs with QUAL ≥ 30 and a total read depth between 40 and 1600.

Because the reference belongs to a different species, SNPs at which all eight cultivars carried the same non-reference genotype represent fixed interspecific differences rather than cultivar variation. These were removed with PLINK v2.00 [37] before distance estimation. Pairwise 1−IBS (identity-by-state) distances were then computed from the remaining cultivar-polymorphic SNPs with PLINK v1.90 [38], and cultivars were clustered from that matrix by UPGMA (average linkage). The correspondence between the chloroplast and nuclear distance matrices was assessed by a Mantel test using Spearman’s rank correlation with 99,999 permutations of cultivar labels, which preserves the non-independence of the 28 pairwise distances. This test and the correlations reported below were implemented in Python with SciPy v1.15.2.

Candidate markers for cultivar identification were selected from the filtered SNPs. A SNP was retained if it had QUAL ≥ 200, a per-sample read depth of 8–60 in all eight cultivars, no missing genotype, a position on one of the seven chromosomes, and no other cultivar-polymorphic site within 60 bp on either side. Coverage of at least 8× was further required at every position of the resulting 121 bp window in every cultivar. A minimal panel whose multi-locus genotype is unique to each cultivar was then assembled greedily. Observed heterozygosity per cultivar was estimated with PLINK v1.90 as the proportion of cultivar-polymorphic SNPs at which the cultivar was heterozygous; this quantity is conditioned on polymorphic sites and is therefore much larger than genome-wide heterozygosity per base pair, which was estimated separately as described below. Three checks were used to test whether the distance estimates reflected genotype rather than data properties. First, 1−IBS distances were compared with Euclidean distances computed from allele counts. Second, mean distance per cultivar was correlated with sequencing depth and with mapping rate. Third, because IBS-based distances depend on heterozygosity, distances were recomputed from the subset of sites at which all eight cultivars were homozygous.

Because mapping to a congeneric reference can collapse paralogous or repetitive regions onto one reference copy and generate spurious heterozygous calls, the heterozygous calls were examined for the signatures of such collapse: an excess of heterozygous calls at sites of low mapping quality, at sites of unusually high depth, and with skewed allelic balance. The proportion of calls that were heterozygous was tabulated by site mapping quality (INFO/MQ), by per-sample depth, and by the alternate-allele fraction of the call (FORMAT/AD). A strict SNP set was then defined by retaining only sites with MQ ≥ 50, a per-sample depth of 8–40× in every cultivar (approximately 0.4–2× the mean), an alternate-allele fraction of 0.2–0.8 at every heterozygous call, and no missing genotype, and the distance, clustering and Mantel analyses were repeated on this set. Genome-wide heterozygosity per base pair was estimated from the strict set as the number of heterozygous calls divided by the number of reference bases covered at 8–40× in that cultivar (bedtools v2.27.1 genomecov), and compared with k-mer estimates for sequenced *Rubus* genomes [39,40]. Because this estimate still depends on the reference, heterozygosity was also estimated without a reference, as in those studies, from the 21-mer spectrum of the trimmed read pairs of each cultivar (Jellyfish v2.3.1 [41]) fitted with GenomeScope 2.0 (v2.1.0) [42] under a diploid model, excluding k-mers with coverage above 2000.

## 3. Results

### 3.1. Chloroplast Genome Structure and Gene Content

The complete cp genomes of eight *R. crataegifolius* cultivars ranged from 155,664 to 155,770 bp in length, differing by a maximum of 106 bp (Table 1). Each genome exhibited the typical angiosperm quadripartite structure consisting of an LSC region (85,374–85,430 bp), an SSC region (18,718–18,778 bp), and a pair of IR regions (25,781–25,786 bp each). The overall GC content was 37.10–37.12%, and all eight genomes contained an identical set of 130 genes (eighty-five protein-coding, thirty-seven tRNA, eight rRNA), with 16 genes duplicated in the IR regions (Figure 1; Table 1 and Table S2). Genome size differences among cultivars were attributable to length variation in the LSC and SSC regions, while IR lengths were highly stable (25,781–25,786 bp). The IR boundaries were highly conserved across all eight cultivars: *rps19* lay entirely within the LSC and *rpl2* entirely within the IRb, its nearest end 40–45 bp from JLB, a functional *ycf1* spanned JSA, and *trnH*-GUG began the LSC at JLA. Only two minor differences distinguished cultivar groups: the *rpl2* distance from JLB/JLA was 45 bp in ‘Dongakheuk’, ‘Dongakhong’, ‘Geumdongwang’, and ‘Hwangeumboll’ versus 40 bp in the remaining four cultivars, and the *ndhF*–JSB distance was 16 bp in seven cultivars but 44 bp in ‘Daesung’ (Figure S1).

### 3.2. Repeat Elements and Codon Usage

A total of 69–82 SSRs were identified per cultivar (Table 2, Figure 2a). Mononucleotide repeats formed the most abundant class (41–54; 62.7% of all SSRs), followed by dinucleotide (12–14; 17.1%), tetranucleotide (9–11; 13.7%), and trinucleotide repeats (4–5; 5.9%); pentanucleotide SSRs were detected only in ‘Dongakheuk’, ‘Dongakhong’, ‘Geumdongwang’, and ‘Hwangeumboll’ (one each), and no hexanucleotide SSR met the minimum of three repeat units (Figure 2b). Among pure mononucleotide SSRs, A/T motifs accounted for 94.1–97.4%, and 8–14 SSRs per cultivar occurred in compound formation. Tandem repeats numbered 41–45 per cultivar, with the 20–39 bp length class being the most frequent (51.4%; Figure 2c). Dispersed repeats ranged from 17 to 23 per cultivar, dominated by forward and palindromic types (Table 2, Figure 2d). Reverse-type dispersed repeats were detected only in ‘Dongakheuk’, ‘Dongakhong’, ‘Geumdongwang’, and ‘Hwangeumboll’, and most dispersed repeats were 30–39 bp long (Figure 2e). Relative synonymous codon usage was virtually identical among the eight cultivars and showed a strong A/T-ending bias, with 70.7% of the codons having RSCU > 1 ending in A or T (Figure S2). Analyzed with identical parameters, the twelve published *Rubus* plastomes contained 62–83 SSRs, 38–68 tandem repeats, and 17–29 dispersed repeats (Table S7); the cultivar values (69–82, 41–45 and 17–23) lie within these ranges, and the previously published *R. crataegifolius* plastome NC_039704 (83, 43, and 17) is indistinguishable from them. Of the 76 SSR loci called in at least one cultivar, 43 varied in length among the eight cultivars (Table S8). Their combined length profiles defined exactly five genotypes, coinciding with the five plastome haplotypes: every cultivar pair with an identical plastome was also identical at all polymorphic SSR loci.

### 3.3. Intraspecific Sequence Variation

A total of 526 variants were identified among the eight cp genomes, comprising three hundred and eighty-nine SNPs, one hundred and thirty-four InDels, and three complex variants. Variant density was highest in the SSC (5.24/kb), followed by the LSC (4.67/kb), and markedly lower in the IR regions (IRb: 0.58/kb; IRa: 0.54/kb) (Figure 3a). Among genic variants, *ycf1* harbored the most variants (29), followed by *rpoC2* (14), *ndhF* (7), *ndhK* (4), *rps3* (4), *ycf2* (4), and *ccsA* (4). Overall, 78.3% of all variants were located in non-coding regions. After normalizing for the length of each partition, the substitution rate in protein-coding sequences (114 variants; 1.45/kb) was 0.23× that of non-coding regions (412 variants; 6.32/kb), and no variants were detected across the 11.9 kb of tRNA and rRNA genes (Figure 4a). Among the 79 protein-coding genes shared by all eight cultivars, 37 were invariant, while the remaining 42 accumulated 73 synonymous and 37 non-synonymous variable sites (N/S = 0.51; Table S3). Pairwise Ka/Ks values calculated from concatenated coding-sequence alignments were far below one in all twenty-two cultivar pairs for which Ks > 0 (median 0.133; range 0.063–0.171), and the upper bound of the bootstrap confidence interval remained below one for every pair (largest upper bound 0.347; Figure 4b, Table S4). The same pattern held in interspecific comparisons of ‘Dongakhong’ with *R. takesimensis* (Ka/Ks = 0.068, 95% CI 0.036–0.117), *R. coreanus* (0.146, 0.120–0.174), and *R. chingii* (0.160, 0.129–0.193), and no individual gene exceeded one in any comparison (Figure 4c; Table S5).

Pairwise SNP analysis identified two haplotype groups and three individually distinct cultivars (Figure 3c): Group A (‘Dongakheuk’, ‘Dongakhong’, ‘Geumdongwang’; zero pairwise SNPs), Group B (‘Dabok’, ‘Geumdongcheon’; zero SNPs), with ‘Daesung’, ‘Goldmoon’, and ‘Hwangeumboll’ each possessing unique haplotypes. ‘Goldmoon’ was closest to Group B (four SNPs), ‘Hwangeumboll’ to Group A (fifty-four SNPs), and ‘Daesung’ was intermediate (eighty-five SNPs to Group B, 309 to Group A). The maximum pairwise distance was 326 SNPs (‘Goldmoon’ vs. ‘Hwangeumboll’).

Among private variants (Figure 3b), ‘Daesung’ exhibited the most (36 SNPs, 19 InDels), with private substitutions distributed across multiple functional genes including *ndhF* (three SNPs + one InDel), *rpoC2* (2), *petB* (2), *ycf2* (2), *rpoB* (1), *psaB* (1), and *ndhD* (1). ‘Hwangeumboll’ had the second-highest number of private variants (28 SNPs, 16 InDels), with genic variants concentrated in *ycf1* (3 SNPs) and *petA* (1 SNP), while the majority (40 of 44) were in intergenic regions. ‘Goldmoon’ possessed only three private SNPs and three private InDels, all in intergenic regions. The remaining five cultivars (‘Dongakheuk’, ‘Dongakhong’, ‘Geumdongwang’, ‘Dabok’, ‘Geumdongcheon’) had no private variants, consistent with their membership in identical-sequence haplotype groups.

### 3.4. Phylogenetic Relationships

The ML tree based on 20 *Rubus* accessions with *Prunus mume* as outgroup resolved two principal lineages within *Rubus* (Figure 5). One (96.9/72) comprised *R. phoenicolasius*, *R. parvifolius*, *R. coreanus*, *R. arcticus,* and *R. buergeri*; the other (100/100) contained all remaining species. Within the latter, all *R. crataegifolius* accessions together with *R. takesimensis* formed a clade (100/100) whose sister was *R. trifidus* (100/100). That group was in turn sister to a clade (100/100) in which *R. hirsutus* was sister to *R. sumatranus* plus *R. chingii* + *R. corchorifolius* (79.6/82 and 100/100, respectively). *R. trifidus* therefore fell outside the *R. crataegifolius* clade rather than within the remainder of the genus.

Within the *R. crataegifolius*–*R. takesimensis* clade, two subclades were recovered, both with maximal support. The first (100/100) contained the three Group-A cultivars ‘Dongakheuk’, ‘Dongakhong’, and ‘Geumdongwang’, whose branch lengths were effectively zero in keeping with their identical plastomes (99.9/100), together with ‘Hwangeumboll’. The second (100/100) comprised ‘Daesung’ with *R. takesimensis* (99.9/100) on one side and, on the other (100/100), ‘Goldmoon’ together with the Group B cultivars ‘Dabok’ and ‘Geumdongcheon’ (76/84) and the published *R. crataegifolius* plastome NC_039704; relationships among these last three were poorly supported (0/36). ‘Daesung’ was thus placed with *R. takesimensis* rather than with any other *R. crataegifolius* accession.

The minimum spanning network of the seven plastome haplotypes (Figure 6) summarizes the same structure without imposing bifurcation. Haplotype group A and ‘Hwangeumboll’ (54 SNPs apart) form one cluster; group B, ‘Goldmoon’ and NC_039704 (each 4 SNPs from group B) form another; and ‘Daesung’ connects the two, lying 84 SNPs from group B, 43 from *R. takesimensis* and 304 from group A. *R. takesimensis* is thus a terminal haplotype attached to ‘Daesung’, not a hub from which the cultivar haplotypes radiate, and the 43 differences between the two exclude the possibility that the ‘Daesung’ sample is a mislabeled *R. takesimensis*.

**Figure 6:**
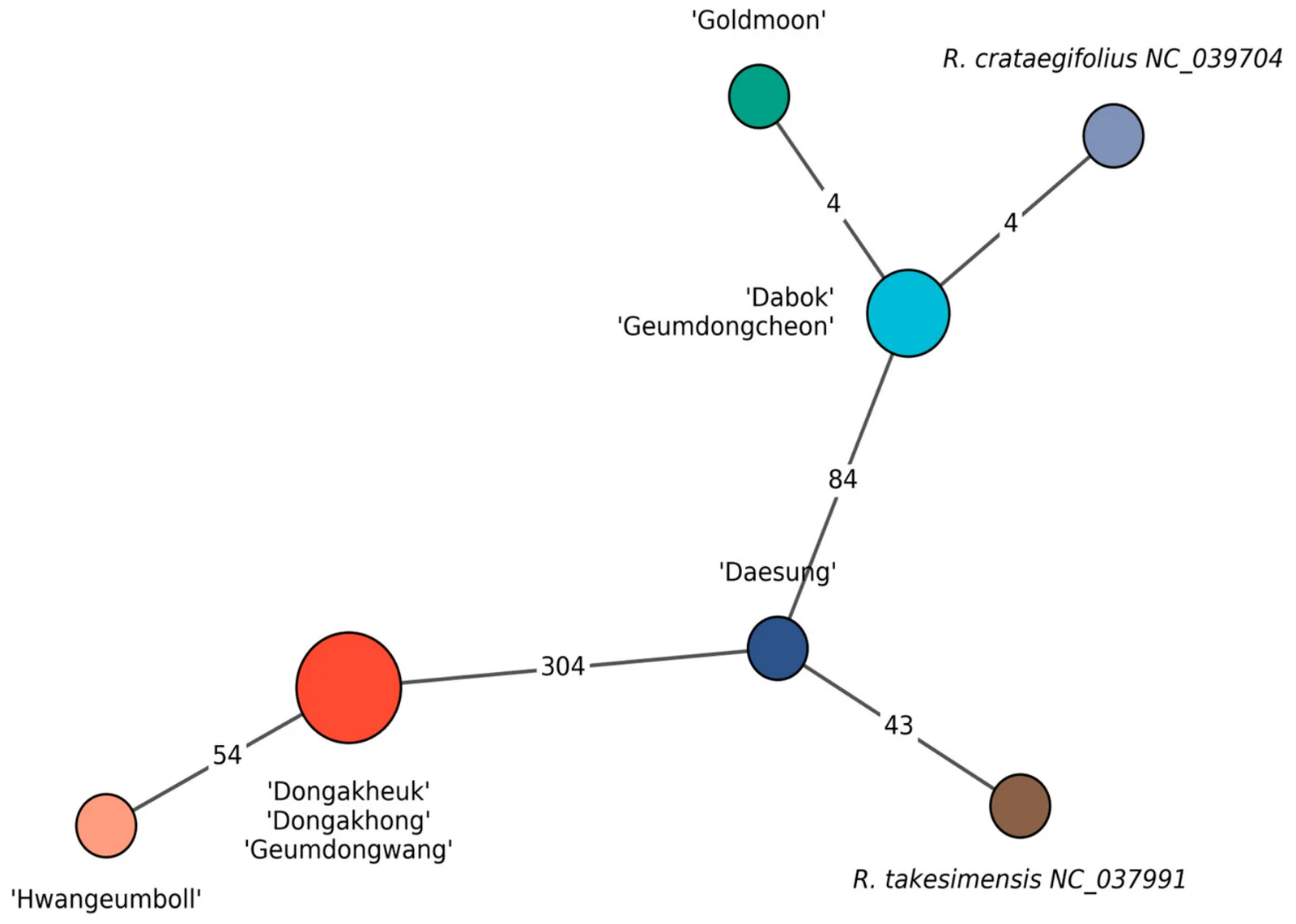
Minimum spanning network of plastome haplotypes among the eight *R. crataegifolius* cultivars, the published *R. crataegifolius* plastome NC_039704, and *R. takesimensis* NC_037991. Circles are haplotypes, scaled to the number of sequences they contain and colored by chloroplast haplotype group as in Figure 7; numbers on edges are SNP differences between haplotypes over the 155,357 alignment columns free of gaps and ambiguous bases in all ten sequences. Edge lengths are not to scale.

**Figure 7:**
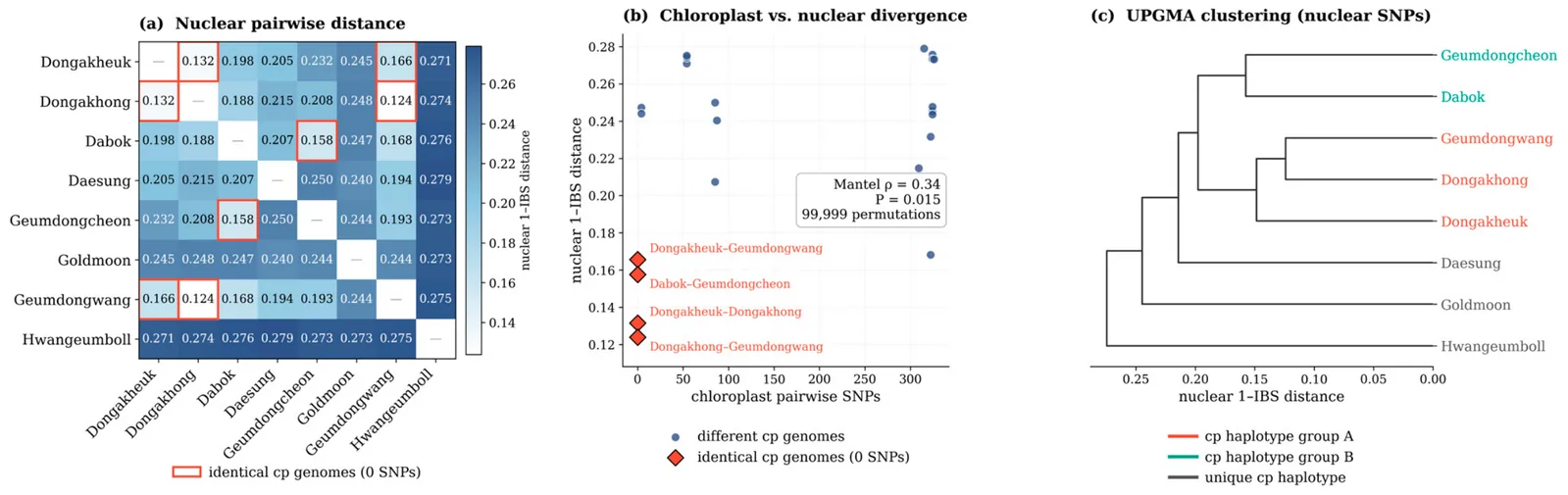
Nuclear SNP-based relationships among the eight *R. crataegifolius* cultivars. (**a**) Matrix of pairwise 1−IBS distances calculated from 4,872,213 cultivar-polymorphic nuclear SNPs; red outlines mark the four cultivar pairs whose chloroplast genomes are identical. (**b**) Chloroplast pairwise SNP distance plotted against nuclear 1−IBS distance for all 28 cultivar pairs, with the Mantel correlation between the two distance matrices; red diamonds denote the pairs with identical chloroplast genomes. (**c**) UPGMA clustering based on the nuclear 1−IBS distances, with cultivar labels colored by chloroplast haplotype group.

### 3.5. Nuclear Genetic Relationships Among Cultivars

Between 90.5% and 91.6% of reads per cultivar mapped to the *R. idaeus* reference at a mean depth of 19.2–21.6×. Joint calling yielded 14,021,997 raw variants, of which 8,308,205 biallelic SNPs passed filtering. Of these, 3,435,992 were monomorphic among the cultivars and therefore represent fixed differences from the reference species, leaving 4,872,213 cultivar-polymorphic SNPs for distance estimation. Every cultivar pair was separated at the nuclear level, with 1−IBS distances ranging from 0.1239 (‘Dongakhong’–‘Geumdongwang’) to 0.2791 (‘Daesung’–‘Hwangeumboll’) (Figure 7a). Critically, the four cultivar pairs whose chloroplast genomes were identical were nevertheless resolved: 0.1315 for ‘Dongakheuk’–‘Dongakhong’, 0.1656 for ‘Dongakheuk’–‘Geumdongwang’, and 0.1239 for ‘Dongakhong’–‘Geumdongwang’ within haplotype group A, and 0.1576 for ‘Dabok’–‘Geumdongcheon’ within group B (Figure 7b). These four were also the most similar pairs genome-wide, indicating that the chloroplast haplotype groups reflect genuine relatedness that the plastome alone cannot resolve. Their separation is far larger than any plausible genotyping error. Of the 2,442,205 sites genotyped in all eight cultivars, the two most similar cultivars (‘Dongakhong’ and ‘Geumdongwang’) differed at 550,557 sites (22.5%), and the other three chloroplast-identical pairs differed at 594,254 to 790,414 sites (24.3–32.4%).

Chloroplast and nuclear divergence were discordant in both directions. The three cultivars of haplotype group A share an identical plastome, yet their nuclear distances span 0.1239–0.1656, which is 27% of the entire range observed across all 28 cultivar pairs. Conversely, ‘Goldmoon’ differs from ‘Dabok’ and ‘Geumdongcheon’ by only four chloroplast SNPs but is among the most distant cultivars from them in nuclear terms (1−IBS = 0.244–0.247). This is the pattern expected when cultivars share a recent maternal ancestor but divergent paternal backgrounds. Across the two full matrices the measures were correlated, but weakly (Mantel ρ = 0.34, *p* = 0.015; Figure 7b); with only eight cultivars this test has limited power, and we treat it as descriptive rather than as the primary evidence. UPGMA clustering of the nuclear distances (Figure 7c) recovered group B (‘Dabok’ and ‘Geumdongcheon’) and placed the three group-A cultivars together. It separated ‘Goldmoon’ from group B, however, and resolved ‘Hwangeumboll’ as the most divergent cultivar (1−IBS = 0.271–0.279 from all others), in contrast to its intermediate position in the plastome analysis (54 SNPs from group A).

Observed heterozygosity, expressed as the proportion of the 4,872,213 cultivar-polymorphic SNPs at which a cultivar was heterozygous, ranged from 0.403 (‘Goldmoon’) to 0.443 (‘Dongakheuk’). The distance estimates were not explained by the amount or quality of data. Mean 1−IBS distance per cultivar showed no detectable relationship with sequencing depth (r = −0.04, *p* = 0.93) or mapping rate (r = −0.04, *p* = 0.92), although with eight cultivars only strong effects would be detectable. Euclidean distances computed from allele counts gave an identical rank ordering of all 28 pairs (Spearman ρ = 1.000). Because IBS-based distances depend on heterozygosity, and mean distance was negatively correlated with heterozygosity across cultivars (r = −0.63, *p* = 0.10), distances were also recomputed from the 54,829 sites at which all eight cultivars were homozygous. This subset preserved the overall structure (ρ = 0.86 with the full data) and again placed ‘Hwangeumboll’ and ‘Goldmoon’ as the most divergent cultivars. The nearest neighbor of ‘Geumdongwang’ changed from ‘Dongakhong’ to ‘Dabok’, however, indicating that the finest distinctions among the closest cultivars are sensitive to the site set used.

The heterozygous calls carried the expected signature of collapsed paralogues: the proportion of calls that were heterozygous was 0.43–0.51 at sites with MQ < 50 but 0.14–0.16 at MQ = 60, and 0.53–0.58 at per-sample depths above 40× (about twice the mean) against 0.19–0.35 at 8–40×, and the alternate-allele fraction at heterozygous calls had a shoulder below 0.2 (Figure S3). A strict set (MQ ≥ 50, depth 8–40× in every cultivar, allele balance 0.2–0.8, no missing genotype) retained 1,811,810 cultivar-polymorphic SNPs (37% of the original); on it, observed heterozygosity fell to 0.279–0.331 and heterozygosity per base pair over the 121.5–122.9 Mb covered at 8–40× was 0.41–0.49%. The reference-free estimate was higher, as expected for a measure that includes the divergent half of the genome: every 21-mer spectrum showed a heterozygous peak at 10–12× and a homozygous peak at twice that coverage, and GenomeScope gave heterozygosity of 1.50% (‘Goldmoon’) to 1.83% (‘Daesung’) and a haploid genome length of 238–261 Mb (Table S9, Figure S4), slightly above the 1.54% of *R. idaeus* ‘Autumn Bliss’ and well above ‘Malling Jewel’ (0.45%) [39] and *R. chingii* (0.80%) [40]. The observed heterozygosity reported above is therefore what a genuinely heterozygous outcrossing genome yields when the statistic is conditioned on polymorphic sites, not a product of the reference, although a fraction of the individual calls was. The conclusions were unchanged on the strict set: every cultivar pair remained separated (1−IBS 0.104–0.325), the four chloroplast-identical pairs were again the four smallest distances (0.104–0.161), the 28 distances were rank-correlated at ρ = 0.986 with the original ones, the UPGMA topology was identical (only ‘Goldmoon’ exchanged its two nearest neighbors, which lay within 0.003 of each other), mean distance was unrelated to depth (r = −0.04, *p* = 0.93) or heterozygosity (r = 0.12, *p* = 0.79), and the Mantel correlation with the chloroplast matrix was slightly stronger (ρ = 0.38, *p* = 0.003).

As a practical corollary, applying the marker selection criteria described above to the filtered SNPs yielded a set of five SNPs (RcNu-01 to RcNu-05) whose combined genotypes are unique to each of the eight cultivars; details, genotypes and flanking sequences are given in Table S6 and Note S1. All five SNPs are retained in the strict set described above, and the heterozygous calls on which RcNu-04 and RcNu-05 depend have alternate-allele fractions of 0.50–0.60 at 20–26× depth. Because the SNPs were selected on the same eight cultivars that they are then shown to separate, this is an internal demonstration rather than a validated assay, and its extension to other material remains to be tested.

## 4. Discussion

The cp genome features of the eight *R. crataegifolius* cultivars are consistent with previously reported *R. crataegifolius* [11] and other *Rubus* species [13,14]. The size variation of only 106 bp is comparable to or smaller than intraspecific variation in other Rosaceae [43], confirming strong structural constraints on the plastome within this species.

Despite this conservation, the 526 variants identified revealed clear patterns of intraspecific differentiation. The higher variant density in single-copy (SC) regions compared to IR regions is consistent with the copy-correction mechanism that maintains IR sequence homogeneity [44]. The most variable genes—*ycf1*, *rpoC2*, and *ndhF*—are commonly identified as hypervariable loci in angiosperm cp genomes [10]. The concentration of variants in non-coding regions is not in itself evidence of selection, because the coding and non-coding partitions differ in length. After length normalization, however, coding sequences accumulated variants at only 0.23× the non-coding rate, and no variant at all was recovered from the tRNA and rRNA genes. Ka/Ks values were well below one both among the cultivars (median 0.133) and against three congeners (0.068–0.160), with bootstrap confidence intervals excluding one in every comparison and no individual gene exceeding one. The plastid coding sequences of *R. crataegifolius* therefore evolve under strong purifying selection, and no plastid gene shows evidence of positive selection at this taxonomic scale.

The two haplotype groups (Group A and Group B) and the three individually distinct cultivars were recovered consistently by pairwise SNP distances and by the plastome phylogeny. The IR boundary characters were compatible with them, although the *rpl2* character groups ‘Hwangeumboll’ with Group A rather than distinguishing the two. The identical cp genomes within Group A and Group B likely reflect derivation from the same maternal lineage or a recent common maternal ancestor. Since the cp genome is maternally inherited in most angiosperms, these groupings provide insight into maternal lineage relationships, although documented breeding records would be needed to confirm the specific seed parents involved. The IR boundary variation in *rpl2* distance (45 bp vs. 40 bp at JLB/JLA) and the unique *ndhF* distance in ‘Daesung’ (44 bp vs. 16 bp at JSB) represent minor expansion or contraction events, consistent with the gradual boundary shifts described in Rosaceae [43]. They serve as additional phylogenetically informative characters at the intraspecific level.

The nuclear resequencing data address the principal limitation of plastome-based cultivar comparison directly. All four cultivar pairs that were indistinguishable by chloroplast sequence were separated by nuclear SNPs. The identical plastomes of haplotype groups A and B therefore reflect a shared maternal lineage rather than genetic identity, and whole-plastome sequencing alone is not sufficient for cultivar identification in *R. crataegifolius*. At the same time, these four pairs had the smallest nuclear distances of all 28 pairs, so the chloroplast haplotype groups are not artefacts: they identify genuinely close cultivars at a resolution the plastome cannot refine. It is also notable that ‘Goldmoon’, the only cultivar in the set developed by a different institution, was among the two most nuclear-divergent cultivars, as would be expected of a distinct breeding background. The most divergent cultivar overall, ‘Hwangeumboll’, nevertheless came from the same program as the remaining six, so institutional origin alone does not predict nuclear distance.

The plastome is not uninformative. The correlation between chloroplast and nuclear divergence is weak (Mantel ρ = 0.34, *p* = 0.015; ρ = 0.38 on the strict SNP set). With eight cultivars there are only 28 pairwise distances and 8! = 40,320 distinct relabelings, so the test has little power and its *p* value should not be over-interpreted in either direction; we therefore rest the argument on the individual mismatches rather than on the coefficient. Because the plastid genome is maternally inherited in most angiosperms and effectively non-recombining, cultivars sharing a maternal lineage retain an identical plastome regardless of how divergent their pollen parents were. Two of our observations are expected consequences of this: the spread of nuclear distances within haplotype group A, which covers 27% of the range seen across all cultivar pairs, and the pairing of a four-SNP chloroplast difference with one of the largest nuclear distances in ‘Goldmoon’. The same reasoning tempers the plastome tree. The position of ‘Hwangeumboll’, intermediate in the chloroplast analysis but the most divergent cultivar in nuclear terms, illustrates that plastome topology need not track overall genomic relatedness among cultivars of a single species. The same decoupling has been reported between crop species in cultivated *Vaccinium* [16]; the present data show that it also operates among cultivars within a single species, where the plastome differences available to resolve them are correspondingly smaller.

The placement of ‘Daesung’ with *R. takesimensis* (SH-aLRT 99.9/UFBoot 100) rather than within any other *R. crataegifolius* sub-clade is the most striking feature of the plastome tree. *R. takesimensis* is an anagenetically derived species endemic to Ulleung Island, Korea, thought to have differentiated from a *R. crataegifolius* ancestor in isolation [12]. The topology is thus consistent with the two sharing a more recent maternal ancestor than either does with the remaining cultivars, but a shared plastome lineage can arise in several ways. A substituted or mislabeled *R. takesimensis* sample is excluded—the ‘Daesung’ plastome differs from NC_037991 at 43 sites and carries 55 private variants—and its unremarkable nuclear distances and heterozygosity give no sign of a recent interspecific hybrid, although with two species this closely related such a sign would be weak in any case (mislabeling at the cultivar level would affect only the name, not the plastome relationship). Two explanations remain: incomplete lineage sorting, with the ‘Daesung’ haplotype belonging to a plastome lineage already present in the *R. crataegifolius* ancestor before *R. takesimensis* differentiated from it, or past introgression (chloroplast capture) of a *takesimensis*-type plastome into the ancestry of ‘Daesung’ [45,46]. The network (Figure 6) does not separate them: *R. takesimensis* is a terminal haplotype attached to ‘Daesung’, as expected under both. Given the cytonuclear discordance documented above, however, this is a statement about the maternal line alone, and we were unable to test it with biparentally inherited markers. No nuclear sequence data suitable for the purpose are publicly available for *R. takesimensis*, and neither of the two nuclear markers that do exist for the species—nrDNA ITS and LEAFY intron 2—proved usable, for reasons set out in Note S2. A recent synthesis of the Ulleung Island endemics reached the same conclusion: evidence for *R. takesimensis* remains almost entirely plastid-based, and its nuclear history is unresolved [47]. Whether the ‘Daesung’ plastome reflects a documented breeding event, introgression from the Ulleung Island lineage, or ancestral polymorphism retained as an unsampled component of natural plastome diversity in *R. crataegifolius* therefore remains open. Distinguishing among these possibilities will require nuclear data from *R. takesimensis* and from wild populations across the species range.

The private plastome variation carried by ‘Daesung’ (36 SNPs, 19 InDels) and ‘Hwangeumboll’ (28 SNPs, 16 InDels) indicates derivation from more divergent maternal lineages than the other cultivars. For ‘Hwangeumboll’ the nuclear data agree; for ‘Daesung’ they do not, its nuclear distances being intermediate among the eight cultivars, so its distinctiveness is largely maternal.

Regarding SSR composition, mononucleotide repeats were the most abundant class (62.7%) and were overwhelmingly A/T-rich (94.1–97.4%), matching the pattern reported for other *Rubus* plastomes analyzed under comparable thresholds [13]. Re-analysis of twelve published *Rubus* plastomes with identical parameters confirmed that the cultivars are unremarkable in repeat content: their SSR, tandem-repeat, and dispersed-repeat counts fall within the ranges of the genus (Table S7), and the published *R. crataegifolius* plastome is indistinguishable from them. We note that a permissive minimum of two repeat units inflates penta- and hexanucleotide counts by an order of magnitude and renders SSR totals incomparable across studies; the conservative minimum of three units applied here yields counts and a class composition directly comparable with the published *Rubus* literature. The SSR loci are nonetheless of some practical value. Forty-three of the 76 loci vary in length among the cultivars (Table S8), mostly as differences of one or two units in A/T mononucleotide runs that are readily typed by capillary electrophoresis, so a plastid SSR assay could assign a sample to one of the five haplotypes without sequencing the plastome. Their limitation is that of the plastome itself: because the SSRs are plastid loci, their multilocus profiles reproduce the five haplotypes exactly and cannot separate the cultivars within haplotype groups A and B. Plastid SSRs therefore complement, but cannot replace, the nuclear markers. The A/T-ending codon usage bias and mononucleotide SSR composition are consistent with general angiosperm plastome patterns [48]. The restriction of reverse-type dispersed repeats to Group A and ‘Hwangeumboll’ may reflect lineage-specific structural features.

Several limitations should be noted. First, one plant was sequenced per cultivar. For clonally propagated material this is the usual design, but we cannot exclude somatic variation or mislabeled stock within a cultivar, and the genotypes reported here, including those of the marker panel, are those of the sampled plants. Second, although the nuclear SNP data resolve all eight cultivars, breeding records were not available to us, so the maternal and paternal relationships inferred here could not be validated against documented pedigrees. Third, the present study is based entirely on cultivated cultivars; comparative analysis incorporating wild *R. crataegifolius* populations from across the natural range (Korea, China, Japan, Russian Far East) would be necessary to place the observed haplotype structure within a broader population-genetic and biogeographic framework. Fourth, the nuclear analysis relies on mapping to a congeneric reference, because no chromosome-level assembly exists for *R. crataegifolius*. Between 90.5% and 91.6% of reads mapped, but only about 60% of the reference was covered by at least one read and 49% at 8× or more, so the comparison rests on the conserved half of the genome; interspecific fixed differences were removed before distance estimation, but residual reference bias cannot be excluded; regions divergent enough to prevent mapping are necessarily under-represented. Mapping to a congener can also collapse paralogous regions onto a single reference copy and thereby generate spurious heterozygous calls, and the data show that it did: heterozygous calls were concentrated at sites of low mapping quality and of roughly double depth (Figure S3). With eight individuals neither Hardy–Weinberg nor excess-heterozygosity filtering is applicable, so the affected sites were removed by filtering on mapping quality, depth, and allelic balance; the agreement between the original and strict sets (Section 3.5), together with the independence of the distances from depth and mapping rate and their stability on homozygous sites alone, is the evidence that the conclusions do not rest on paralogue-derived calls, and the reference-free k-mer estimate shows that the high heterozygosity itself is real. Fifth, the eight-cultivar sample size precludes population-genetic estimators that depend on allele frequencies, so we restricted the analysis to pairwise distances and clustering. IBS-based distances are moreover sensitive to heterozygosity; as noted above the ranking of cultivars survived recomputation from homozygous sites alone, but the nearest-neighbor relationships among the most similar cultivars did shift, and those finest distinctions should therefore be treated as provisional. Finally, the SNP panel proposed here, like the hypervariable plastid regions identified around *ycf1* and the intergenic spacers, is a computational prediction; conversion into assays and validation on independent plant material remain to be done. By way of comparison, the KASP fingerprinting panel available for *R. idaeus* comprises 48 experimentally validated assays, reducible to 24 [17], and the perilla panel cited above rests on 150 markers validated across 48 cultivars [49]. The five markers reported here are a starting point for an equivalent resource in *R. crataegifolius*, not a finished one.

## 5. Conclusions

The results support three conclusions that differ in the strength of the evidence behind them. First, plastome haplotypes inform on maternal lineage: the five haplotypes identify which of the eight cultivars share a maternal lineage, including a link between ‘Daesung’ and the *R. takesimensis* plastome, but they cannot identify the seed parent itself without pedigree records, they leave cultivars with a common maternal origin indistinguishable, and their distances are a poor guide to overall genomic relatedness. Second, genome-wide nuclear SNPs discriminate all eight cultivars analyzed, a result that survived stringent filtering of heterozygous calls; because one plant was sequenced per cultivar, this is a statement about the plants sampled rather than a validated identification system for the cultivars. Third, the five-SNP panel derived from these data is a candidate tool: it separates the eight cultivars in silico but has not been converted into assays or tested on independent plants, and it should not be regarded as suitable for routine cultivar identification until that has been done. For breeding programs that maintain related cultivars, we therefore recommend pairing plastome haplotyping, which is informative about maternal descent, with validated nuclear markers for identity, rather than treating either as a substitute for the other.

## Figures and Tables

**Figure 1:**
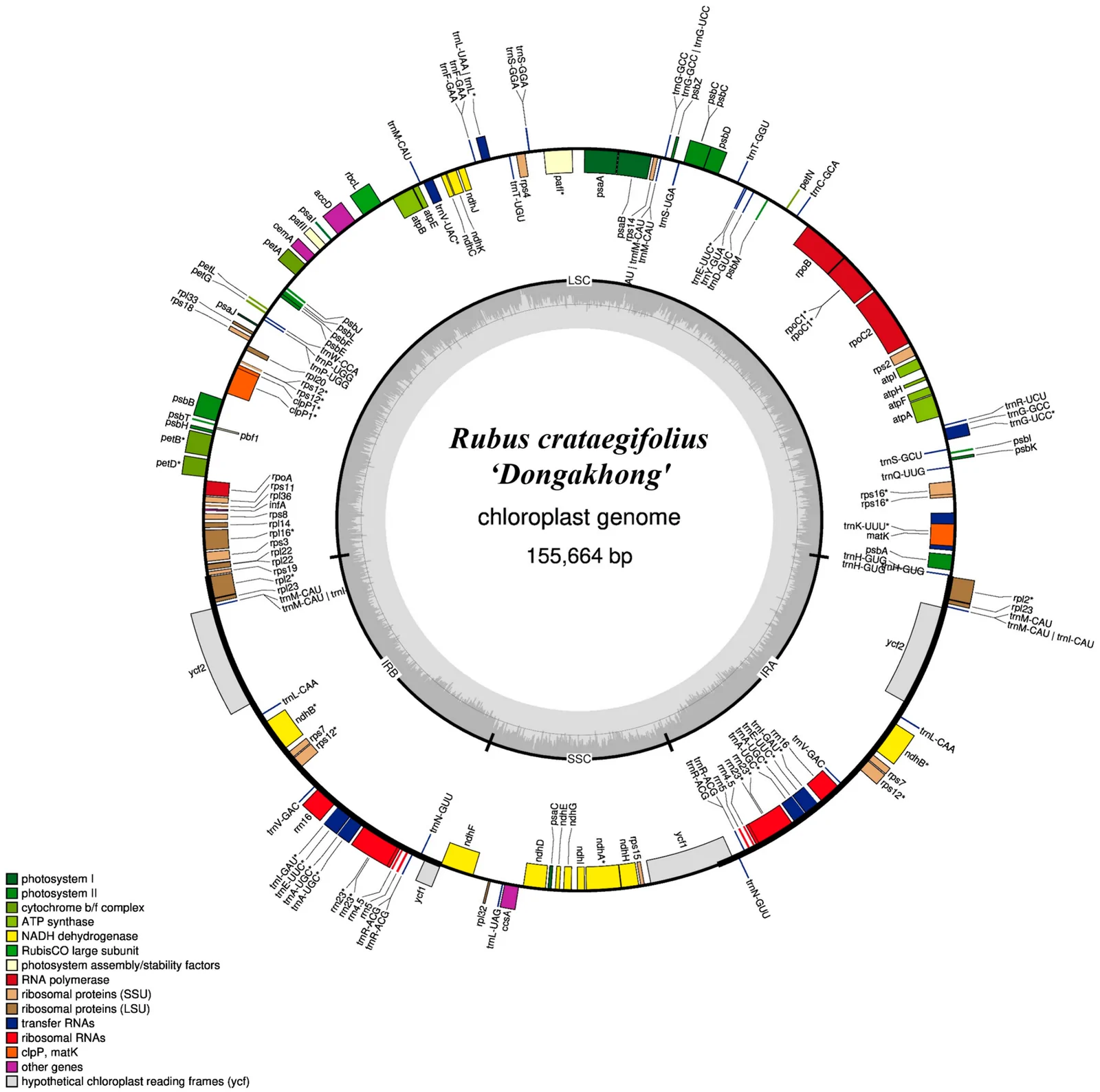
Circular gene map of the *R. crataegifolius* ‘Dongakhong’ chloroplast genome (representative). Genes drawn inside the outer circle are transcribed clockwise, while those outside are transcribed counterclockwise. Genes are color-coded by functional group. The inner circle indicates GC content variation across the genome. Asterisks mark genes that contain introns. LSC, large single-copy region; SSC, small single-copy region; IRa and IRb, inverted repeat regions.

**Figure 2:**
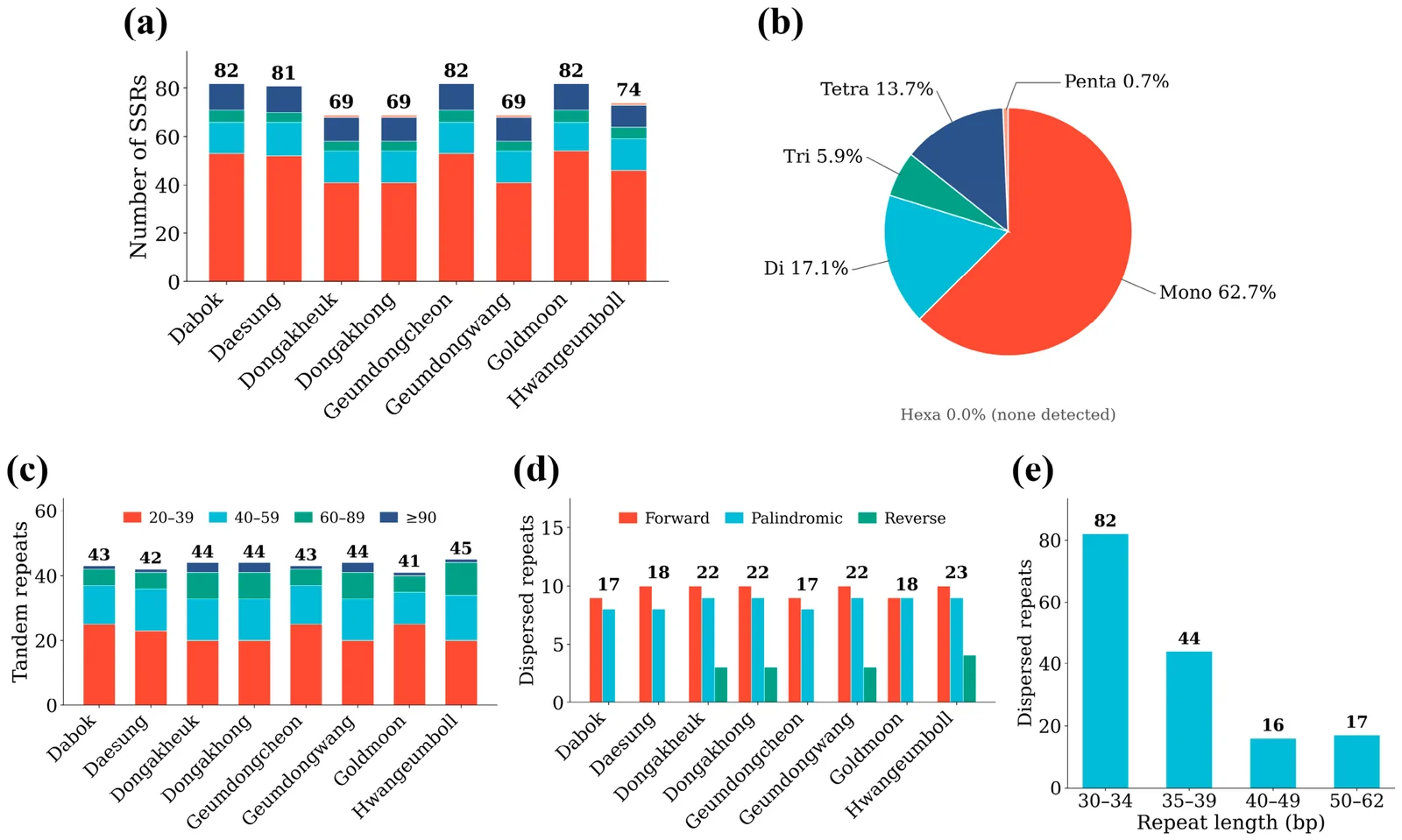
Repeat element composition in the chloroplast genomes of eight *R. crataegifolius* cultivars. (**a**) Number of SSRs per cultivar classified by repeat unit size (mononucleotide to hexanucleotide). (**b**) Average proportional distribution of SSR types across all eight cultivars; percentages are rounded to one decimal place and therefore sum to 100.1%. (**c**) Length distribution of tandem repeats. (**d**) Types of dispersed repeat (forward, palindromic and reverse; complement-type repeats were not detected) per cultivar. (**e**) Size distribution of dispersed repeats. Counts in (**d**,**e**) exclude the IR-derived palindromic repeat (the IRa/IRb pair, 25,781–25,786 bp) present once in every cultivar.

**Figure 3:**
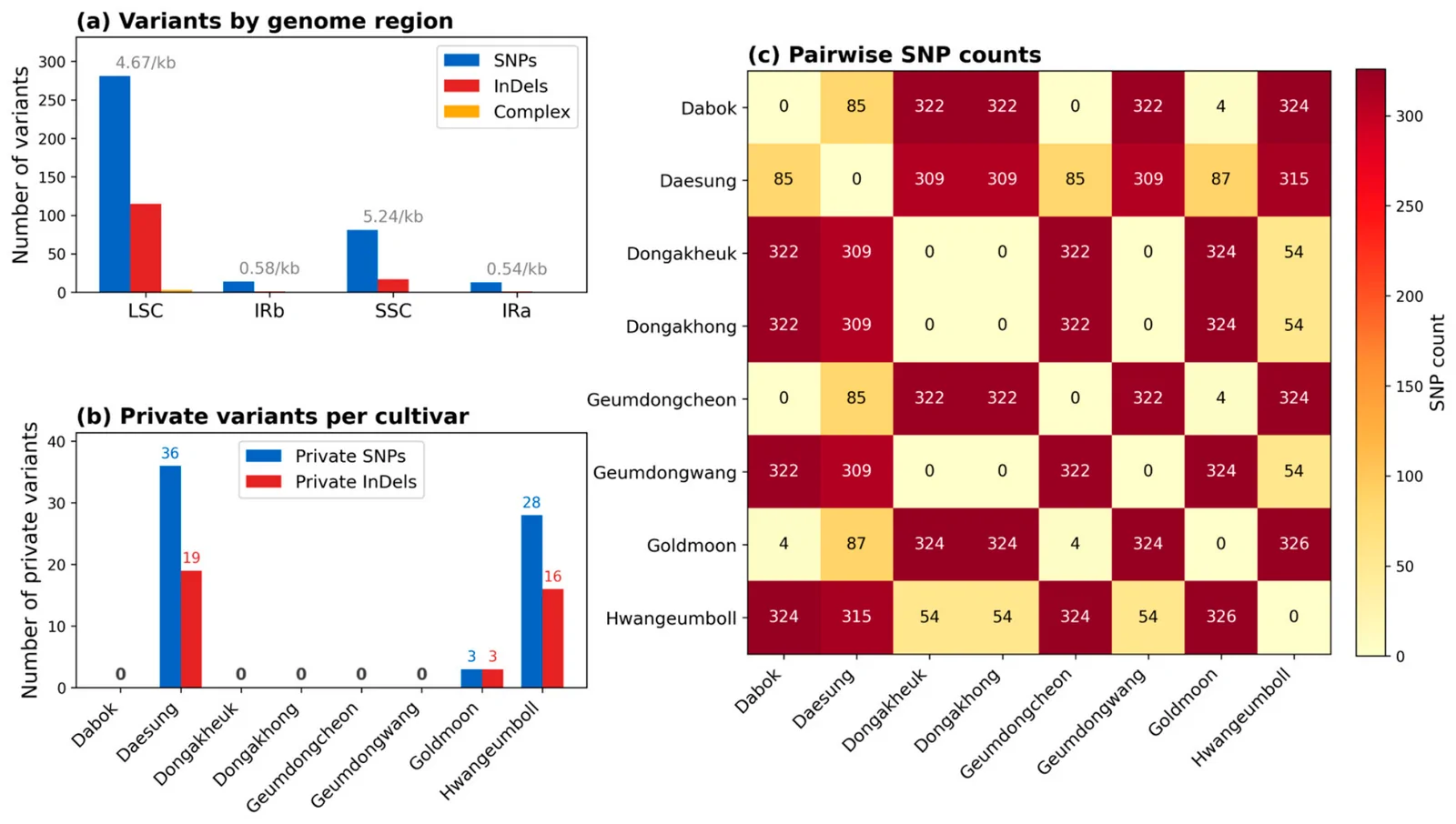
Intraspecific sequence variation among eight *R. crataegifolius* chloroplast genomes. (**a**) Variant density (variants/kb) by genomic region (LSC, SSC, IRa, IRb). (**b**) Number of private variants (SNPs and InDels) per cultivar. (**c**) Pairwise SNP distances among the eight cultivars shown as a heatmap. Two haplotype groups are apparent—Group A (‘Dongakheuk’, ‘Dongakhong’, ‘Geumdongwang’; zero SNPs) and Group B (‘Dabok’, ‘Geumdongcheon’; zero SNPs)—along with three individually distinct cultivars (‘Daesung’, ‘Goldmoon’, ‘Hwangeumboll’).

**Figure 4:**
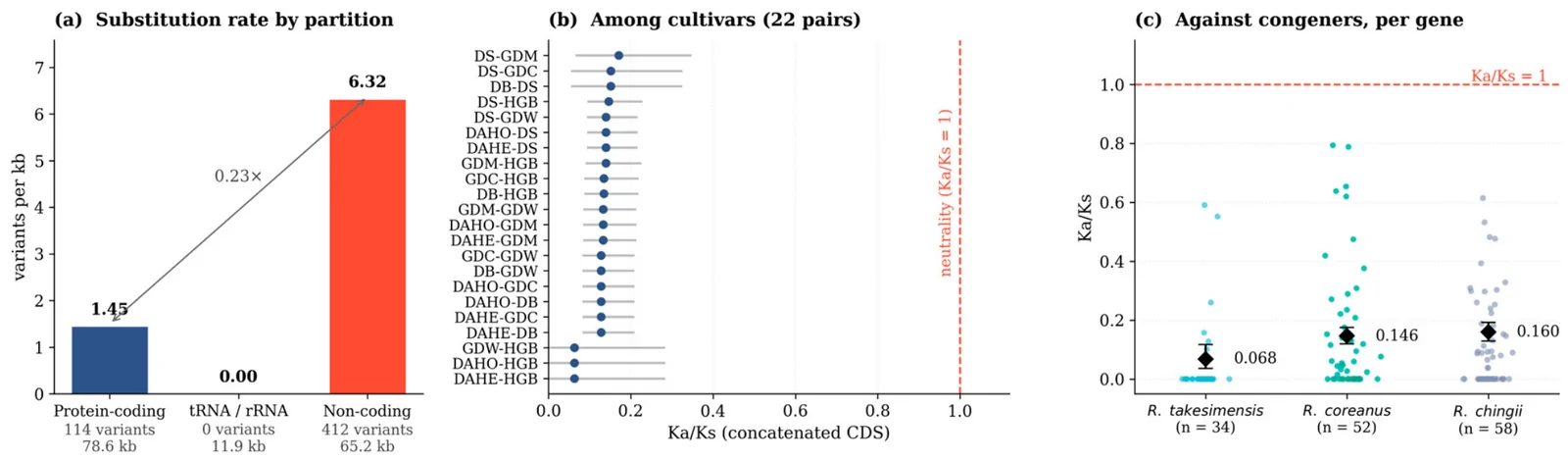
Selection on plastid coding sequences. (**a**) Substitution rate per kb in each genomic partition of the *R. crataegifolius* plastome; the number of variants and the partition length are given below each bar. Coding sequences accumulate variants at 0.23× the non-coding rate and no variant was recovered from the tRNA and rRNA genes. (**b**) Ka/Ks from concatenated coding-sequence alignments for the 22 cultivar pairs with Ks > 0, ordered by value; horizontal bars are 95% bootstrap confidence intervals over codon columns. (**c**) Gene-wise Ka/Ks against three congeners (points), with the concatenated estimate and its 95% confidence interval overlaid (diamonds); n is the number of genes for which Ks > 0. Dashed lines mark Ka/Ks = 1. Cultivar abbreviations used in (**b**): DAHE, Dongakheuk; DAHO, Dongakhong; DB, Dabok; DS, Daesung; GDC, Geumdongcheon; GDM, Goldmoon; GDW, Geumdongwang; HGB, Hwangeumboll.

**Figure 5:**
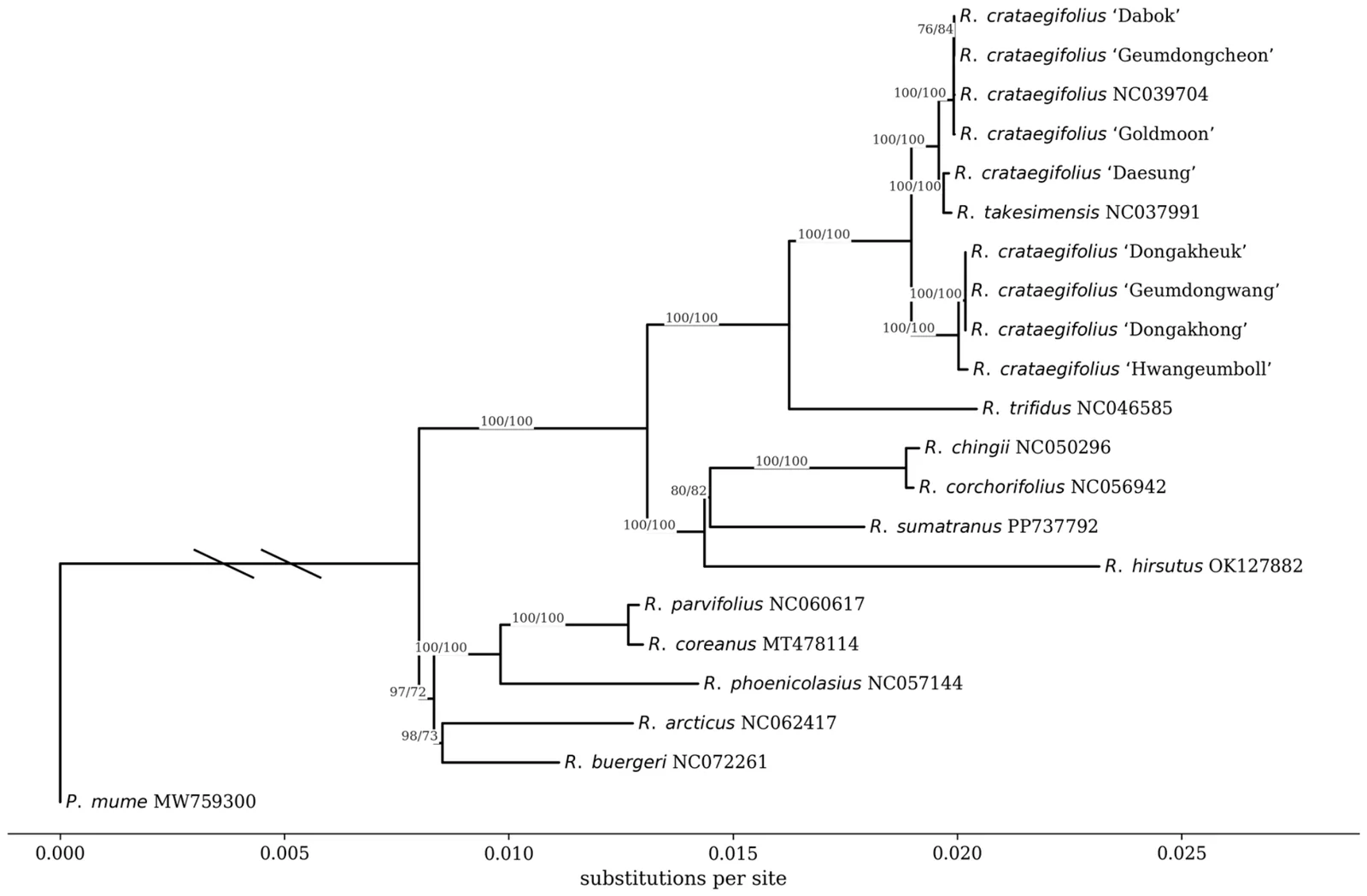
Maximum likelihood phylogeny based on complete chloroplast genome sequences of 20 *Rubus* accessions with *Prunus mume* as outgroup (167,962 aligned positions; TVM+F+R3). Node labels give SH-aLRT/ultrafast bootstrap support (%) from 1000 replicates each; values below 50% are omitted. The branch subtending the ingroup is truncated (double slash) because it is approximately twenty times longer than the divergences within the *R. crataegifolius* clade.

**Table 1:** Chloroplast genome features of eight *R. crataegifolius* cultivars.

Cultivar	GenBank Accession	Genome Size (bp)	LSC (bp)	SSC (bp)	IR (bp)	GC (%)	Genes	CDS	tRNA	rRNA
‘Dabok’	PZ143674	155,713	85,401	18,750	25,781	37.12	130	85	37	8
‘Daesung’	PZ143675	155,770	85,430	18,778	25,781	37.11	130	85	37	8
‘Dongakheuk’	PZ143672	155,664	85,374	18,718	25,786	37.11	130	85	37	8
‘Dongakhong’	PZ143673	155,664	85,374	18,718	25,786	37.11	130	85	37	8
‘Geumdongcheon’	PZ143676	155,713	85,401	18,750	25,781	37.12	130	85	37	8
‘Geumdongwang’	PZ143678	155,664	85,374	18,718	25,786	37.11	130	85	37	8
‘Goldmoon’	PZ143677	155,702	85,390	18,750	25,781	37.12	130	85	37	8
‘Hwangeumboll’	PZ143679	155,703	85,404	18,727	25,786	37.10	130	85	37	8

IR inverted repeat (one copy), CDS protein-coding sequences. Gene counts include IR-duplicated copies.

**Table 2:** Summary of repeat elements in eight *R. crataegifolius* chloroplast genomes.

Cultivar	SSR Total	Mono	Di	Tri	Tetra	Penta	Tandem	Dispersed (F/P/R/C)
‘Dabok’	82	53	13	5	11	0	43	17 (9/8/0/0)
‘Daesung’	81	52	14	4	11	0	42	18 (10/8/0/0)
‘Dongakheuk’	69	41	13	4	10	1	44	22 (10/9/3/0)
‘Dongakhong’	69	41	13	4	10	1	44	22 (10/9/3/0)
‘Geumdongcheon’	82	53	13	5	11	0	43	17 (9/8/0/0)
‘Geumdongwang’	69	41	13	4	10	1	44	22 (10/9/3/0)
‘Goldmoon’	82	54	12	5	11	0	41	18 (9/9/0/0)
‘Hwangeumboll’	74	46	13	5	9	1	45	23 (10/9/4/0)

F forward, P palindromic, R reverse, C complement. Dispersed repeats were identified with minimum length 30 bp and Hamming distance ≤ 3; the IR-derived palindromic repeat (the IRa/IRb pair, 25,781–25,786 bp) present once in every cultivar is excluded. SSR minimum repeat thresholds: mononucleotide-10, dinucleotide-5, trinucleotide-4, tetranucleotide-3, pentanucleotide-3, hexanucleotide-3.

## Data Availability

The eight complete chloroplast genome sequences generated in this study are available in GenBank under accession numbers PZ143672–PZ143679. The raw sequencing reads supporting the reported results are available from the corresponding author upon reasonable request. The custom scripts used for variant detection, codon usage, selection-pressure analysis, and marker selection are available on GitHub (https://github.com/seoljongkim/rubus-crataegifolius-plastome-nuclear, accessed on 4 March 2026) and are archived at Zenodo (https://doi.org/10.5281/zenodo.23364695, accessed on 4 March 2026).

## Supplementary Materials

The following supporting information can be downloaded at: https://www.mdpi.com/article/10.3390/genes17101287/s1, Figure S1: Comparison of inverted repeat (IR) region boundaries among eight *R. crataegifolius* cultivars and two reference sequences (*R. crataegifolius* NC_039704; *R. takesimensis* NC_037991); Figure S2: Relative synonymous codon usage (RSCU) values of 79 protein-coding genes in the *R. crataegifolius* ‘Dongakhong’ chloroplast genome; Figure S3: Quality assessment of nuclear heterozygous calls in the eight *R. crataegifolius* cultivars; Figure S4: GenomeScope 2.0 fits to the 21-mer spectra of the eight *R. crataegifolius* cultivars; Table S1: Published plastome sequences used in the phylogenetic analysis; Table S2: Gene contents of the *R. crataegifolius* chloroplast genome; Table S3: Synonymous and non-synonymous variable sites in the 42 protein-coding genes that varied among the eight *R. crataegifolius* cultivars; Table S4: Pairwise Ka/Ks ratios from concatenated coding-sequence alignments of the eight cultivars; Table S5: Gene-wise Ka/Ks ratios between *R. crataegifolius* ‘Dongakhong’ and three congeners; Table S6: Candidate SNP panel for cultivar identification in *R. crataegifolius*, derived from nuclear resequencing data; Table S7: Repeat content of the eight *R. crataegifolius* cultivars and twelve published *Rubus* plastomes analyzed with identical parameters; Table S8: Length polymorphism of plastid SSR loci among the eight *R. crataegifolius* cultivars; Table S9: Reference-free estimates of heterozygosity and genome size for the eight *R. crataegifolius* cultivars from 21-mer spectra (GenomeScope 2.0); Note S1: Selection and properties of the candidate SNP panel; Note S2: Why the ‘Daesung’–*R. takesimensis* relationship could not be tested with biparentally inherited markers.
