# Supplementary material for: Discordant Chloroplast and Nuclear Divergence Among Eight Rubus crataegifolius (Rosaceae) Cultivars
Source: Genes (Basel). 2026 Oct 15;17(10):1287. doi: 10.3390/genes17101287 (PMC13704536; doi:10.3390/genes17101287)
Supplement: Supplementary file 1 [file genes-17-01287-s001.zip › genes-4625184-supplementary.pdf]

Supplementary Materials

Table S1. Published plastome sequences used in the phylogenetic analysis.

Species	GenBank accession	Role
Rubus crataegifolius	NC_039704	ingroup
Rubus takesimensis	NC_037991	ingroup
Rubus trifidus	NC_046585	ingroup
Rubus hirsutus	OK127882	ingroup
Rubus chingii	NC_050296	ingroup
Rubus corchorifolius	NC_056942	ingroup
Rubus sumatranus	PP737792	ingroup
Rubus phoenicolasius	NC_057144	ingroup
Rubus coreanus	MT478114	ingroup
Rubus parvifolius	NC_060617	ingroup
Rubus arcticus	NC_062417	ingroup
Rubus buergeri	NC_072261	ingroup
Prunus mume	MW759300	outgroup

Together with the eight cultivar plastomes generated in this study these give 20 *Rubus* accessions plus one outgroup. The *Rubus coreanus* accession MT478114 was also used for the interspecific Ka/Ks comparison; *Rubus takesimensis* NC_037991 and *Rubus chingii* NC_050296 were likewise used in both analyses.

Table S2. Gene contents of the *Rubus crataegifolius* chloroplast genome (130 genes: 85 protein-coding, 37 tRNA, 8 rRNA). Genes containing introns, trans-spliced genes, and genes duplicated in the inverted repeat regions are indicated.

Role	Gene category	Gene names	No.
**Photosynthesis**	Photosystem I	*psaA, psaB, psaC, psaI, psaJ*	5
	Photosystem II	*psbA, psbB, psbC, psbD, psbE, psbF, psbH, psbI, psbJ, psbK, psbL, psbM, psbN, psbT, psbZ*	15
	Cytochrome b/f complex	*petA, petB*[1], *petD*[1], *petG, petL, petN*	6
	ATP synthase	*atpA, atpB, atpE, atpF, atpH, atpI*	6
	NADH dehydrogenase	*ndhA*[1], *ndhB*[1*], *ndhC, ndhD, ndhE, ndhF, ndhG, ndhH, ndhI, ndhJ, ndhK*	12
	RuBisCO	*rbcL*	1
	Assembly of photosystem I	*ycf3*[2], *ycf4*	2
**Transcription and translation**	RNA polymerase	*rpoA, rpoB, rpoC1*[1], *rpoC2*	4
	Ribosomal proteins (SSU)	*rps2, rps3, rps4, rps7*[*], *rps8, rps11, rps12*[†], *rps14, rps15, rps16*[1], *rps18, rps19*	14
	Ribosomal proteins (LSU)	*rpl2*[1*], *rpl14, rpl16*[1], *rpl20, rpl22, rpl23*[*], *rpl32, rpl33, rpl36*	11
	Translation initiation factor	*infA*	1
	Ribosomal RNA	*rrn4.5*[*], *rrn5*[*], *rrn16*[*], *rrn23*[*]	8
	Transfer RNA	*trnA-UGC*[1*], *trnC-GCA, trnD-GUC, trnE-UUC, trnF-GAA, trnfM-CAU, trnG-GCC, trnG-UCC*[1], *trnH-GUG, trnI-CAU*[*], *trnI-GAU*[1*], *trnK-UUU*[1], *trnL-CAA*[*], *trnL-UAA*[1], *trnL-UAG, trnM-CAU, trnN-GUU*[*], *trnP-UGG, trnQ-UUG, trnR-ACG*[*], *trnR-UCU, trnS-GCU, trnS-GGA, trnS-UGA, trnT-GGU, trnT-UGU, trnV-GAC*[*], *trnV-UAC*[1], *trnW-CCA, trnY-GUA*	37
**Other**	RNA processing	*matK*	1
	Cytochrome c-type synthesis	*ccsA*	1
	Carbon metabolism	*cemA*	1
	Fatty acid synthesis	*accD*	1
	Proteolysis	*clpP*[2]	1
	Component of TIC translocon	*ycf1*	1
	Hypothetical proteins	*ycf2*[*]	2
**Total**			**130**

[1]Gene contains one intron. [2]Gene contains two introns. [†]Trans-spliced gene. [*]Duplicated in the inverted repeat regions (two copies in the genome).

Table S3. Synonymous and non-synonymous variable sites in the 42 protein-coding genes that varied among the eight *R. crataegifolius* cultivars.

Gene	Length (bp)	Synonymous sites	Non-synonymous sites	N/S
ycf1	5,712	12	16	1.33
rpoC2	4,155	7	6	0.86
ndhF	2,244	3	2	0.67
ndhK	681	3	1	0.33
ccsA	966	1	2	2.00
cemA	690	2	1	0.50
ndhD	1,503	1	2	2.00
ndhH	1,182	3	0	0.00
accD	1,467	2	0	0.00
clpP	591	2	0	0.00
matK	1,512	2	0	0.00
petB	648	2	0	0.00
psbD	1,062	2	0	0.00
psbL	117	2	0	0.00
rpoA	996	2	0	0.00
rpoB	3,213	2	0	0.00
rps15	273	2	0	0.00
rps19	279	2	0	0.00
rps2	711	1	1	1.00
rps3	657	2	0	0.00
rps4	606	1	1	1.00
ycf2	6,834	0	2	inf(S=0)
atpA	1,524	1	0	0.00
atpF	555	1	0	0.00
ndhA	1,092	1	0	0.00
ndhC	363	1	0	0.00
petA	969	1	0	0.00
petD	483	1	0	0.00
psaA	2,253	1	0	0.00
psaB	2,205	1	0	0.00
psaI	114	1	0	0.00
psbB	1,527	1	0	0.00
psbC	1,386	1	0	0.00
psbE	252	1	0	0.00
rbcL	1,428	1	0	0.00
rpl14	369	1	0	0.00
rpl22	429	1	0	0.00
rpl33	201	0	1	inf(S=0)
rpl36	114	1	0	0.00
rpoC1	2,034	0	1	inf(S=0)
rps8	405	0	1	inf(S=0)
ycf4	555	1	0	0.00

Counted as variable codon positions across all eight cultivars. Of the 79 protein-coding genes shared by all cultivars, 37 were invariant and are omitted. Totals: 73 synonymous and 37 non-synonymous sites (N/S = 0.51).

Table S4. Pairwise Ka/Ks ratios from concatenated coding-sequence alignments of the eight cultivars.

Cultivar pair	Aligned length (bp)	Synonymous	Non-synonymous	Ka	Ks	Ka/Ks (95% CI)
DAHE-DAHO	68,193	0	0	0.000000	0.000000	n.d.

Cultivar pair	Aligned length (bp)	Synonymous	Non-synonymous	Ka	Ks	Ka/Ks (95% CI)
DAHE-DB	68,193	61	29	0.000528	0.004117	0.1282 (0.0827–0.2086)
DAHE-DS	68,193	64	33	0.000603	0.004317	0.1397 (0.0930–0.2164)
DAHE-GDC	68,193	61	29	0.000528	0.004117	0.1282 (0.0827–0.2086)
DAHE-GDM	68,193	61	30	0.000546	0.004117	0.1327 (0.0844–0.2133)
DAHE-GDW	68,193	0	0	0.000000	0.000000	n.d.
DAHE-HGB	68,193	9	2	0.000038	0.000596	0.0632 (0.0000–0.2836)
DAHO-DB	68,193	61	29	0.000528	0.004117	0.1282 (0.0827–0.2086)
DAHO-DS	68,193	64	33	0.000603	0.004317	0.1397 (0.0930–0.2164)
DAHO-GDC	68,193	61	29	0.000528	0.004117	0.1282 (0.0827–0.2086)
DAHO-GDM	68,193	61	30	0.000546	0.004117	0.1327 (0.0844–0.2133)
DAHO-GDW	68,193	0	0	0.000000	0.000000	n.d.
DAHO-HGB	68,193	9	2	0.000038	0.000596	0.0632 (0.0000–0.2836)
DB-DS	68,193	15	8	0.000151	0.000994	0.1516 (0.0548–0.3256)
DB-GDC	68,193	0	0	0.000000	0.000000	n.d.
DB-GDM	68,193	0	1	0.000019	0.000000	n.d.
DB-GDW	68,193	61	29	0.000528	0.004117	0.1282 (0.0827–0.2086)
DB-HGB	68,193	58	29	0.000528	0.003917	0.1347 (0.0867–0.2182)
DS-GDC	68,193	15	8	0.000151	0.000994	0.1516 (0.0548–0.3256)
DS-GDM	68,193	15	9	0.000170	0.000994	0.1705 (0.0657–0.3473)
DS-GDW	68,193	64	33	0.000603	0.004317	0.1397 (0.0930–0.2164)
DS-HGB	68,193	61	33	0.000603	0.004117	0.1465 (0.0944–0.2280)
GDC-GDM	68,193	0	1	0.000019	0.000000	n.d.
GDC-GDW	68,193	61	29	0.000528	0.004117	0.1282 (0.0827–0.2086)
GDC-HGB	68,193	58	29	0.000528	0.003917	0.1347 (0.0867–0.2182)
GDM-GDW	68,193	61	30	0.000546	0.004117	0.1327 (0.0844–0.2133)
GDM-HGB	68,193	58	30	0.000546	0.003917	0.1395 (0.0903–0.2257)
GDW-HGB	68,193	9	2	0.000038	0.000596	0.0632 (0.0000–0.2836)

Confidence intervals are 95% percentile intervals from 300 bootstrap replicates over codon columns. n.d., not determined because Ks = 0; this applies to the four cultivar pairs with identical coding sequences (the three Group A pairs and the Group B pair) and to two pairs with one non-synonymous but no synonymous substitution. Ka/Ks was obtained for 22 of 28 pairs (median 0.1327; range 0.0632–0.1705).

Table S5. Gene-wise Ka/Ks ratios between *R. crataegifolius* 'Dongakhong' and three congeners.

Gene	Length (bp)	*R. takesimensis* S/N	*R. takesimensis* Ka/Ks	*R. coreanus* S/N	*R. coreanus* Ka/Ks	*R. chingii* S/N	*R. chingii* Ka/Ks
accD	1,464	2/0	0.0000	–	–	4/5	0.3022
atpA	1,521	1/0	0.0000	13/1	0.0233	8/1	0.0382
atpB	1,476	0/0	n.d.	11/0	0.0000	7/0	0.0000
atpE	399	0/0	n.d.	1/0	0.0000	2/0	0.0000
atpF	552	0/0	n.d.	7/2	0.0758	7/3	0.1122
atpH	243	0/0	n.d.	0/0	n.d.	0/0	n.d.
atpI	741	0/0	n.d.	2/1	0.1519	2/1	0.1519
ccsA	963	1/2	0.5513	3/7	0.6373	4/7	0.4759
cemA	687	2/1	0.1273	6/5	0.2078	5/5	0.2525
clpP	588	1/0	0.0000	3/0	0.0000	2/0	0.0000
infA	144	–	–	–	–	–	–
matK	1,509	2/0	0.0000	15/30	0.4740	9/21	0.3925
ndhA	1,089	1/0	0.0000	7/4	0.1749	7/6	0.2233
ndhB	1,530	0/0	n.d.	–	–	0/0	n.d.
ndhC	360	1/0	0.0000	2/0	0.0000	2/0	0.0000
ndhD	1,500	1/2	0.5898	14/9	0.1340	12/7	0.1817
ndhE	303	0/0	n.d.	0/1	n.d.	0/2	n.d.
ndhF	2,241	2/0	0.0000	21/31	0.3752	16/19	0.3277
ndhG	528	0/0	n.d.	4/2	0.1482	3/0	0.0000
ndhH	1,179	3/0	0.0000	14/2	0.0608	10/3	0.0795
ndhI	513	0/0	n.d.	–	–	2/2	0.2596
ndhJ	474	0/0	n.d.	1/3	0.7875	3/1	0.0864
ndhK	678	3/1	0.1004	9/1	0.0326	5/4	0.2395
petA	966	0/0	n.d.	5/2	0.1185	4/1	0.0742
petB	645	1/0	0.0000	5/1	0.0592	–	–
petD	480	1/0	0.0000	–	–	4/0	0.0000
petG	111	0/0	n.d.	0/0	n.d.	1/0	0.0000

Gene	Length (bp)	***R. takesimensis* S/N**	***R. takesimensis* Ka/Ks**	***R. coreanus* S/N**	***R. coreanus* Ka/Ks**	***R. chingii* S/N**	***R. chingii* Ka/Ks**
petL	93	0/0	n.d.	0/0	n.d.	0/0	n.d.
petN	87	–	–	–	–	–	–
psaA	2,250	1/0	0.0000	19/1	0.0152	9/0	0.0000
psaB	2,202	0/0	n.d.	7/0	0.0000	3/1	0.0939
psaC	243	0/0	n.d.	3/0	0.0000	2/0	0.0000
psaI	111	1/0	0.0000	0/0	n.d.	1/0	0.0000
psaJ	132	0/0	n.d.	0/0	n.d.	0/0	n.d.
psbA	1,059	1/0	0.0000	6/0	0.0000	4/0	0.0000
psbB	1,524	1/0	0.0000	11/1	0.0267	8/1	0.0371
psbC	1,383	–	–	–	–	–	–
psbD	1,059	2/0	0.0000	7/0	0.0000	7/0	0.0000
psbE	249	1/0	0.0000	2/0	0.0000	0/0	n.d.
psbF	117	0/0	n.d.	1/0	0.0000	2/0	0.0000
psbH	222	0/0	n.d.	1/0	0.0000	0/0	n.d.
psbI	108	0/0	n.d.	0/0	n.d.	2/0	0.0000
psbJ	120	0/0	n.d.	0/0	n.d.	1/0	0.0000
psbK	183	0/0	n.d.	1/1	0.2704	0/0	n.d.
psbL	114	2/0	0.0000	1/0	0.0000	1/0	0.0000
psbM	102	0/0	n.d.	0/0	n.d.	1/0	0.0000
psbN	129	0/0	n.d.	0/0	n.d.	0/0	n.d.
psbT	99	0/0	n.d.	0/0	n.d.	0/0	n.d.
psbZ	186	0/0	n.d.	0/0	n.d.	0/0	n.d.
rbcL	1,425	1/0	0.0000	5/11	0.6529	1/1	0.2982
rpl14	366	1/0	0.0000	2/1	0.1539	1/0	0.0000
rpl16	405	0/0	n.d.	–	–	–	–
rpl2	822	–	–	1/2	0.6191	2/2	0.3085
rpl20	351	0/0	n.d.	6/3	0.1399	0/1	n.d.
rpl22	426	1/0	0.0000	–	–	3/2	0.1880
rpl23	279	0/0	n.d.	0/0	n.d.	0/0	n.d.
rpl32	156	0/0	n.d.	–	–	2/0	0.0000
rpl33	198	1/1	0.2593	1/3	0.7929	1/2	0.5317
rpl36	111	1/0	0.0000	3/0	0.0000	2/0	0.0000
rpoA	993	2/0	0.0000	9/12	0.2348	4/13	0.4814
rpoB	3,210	2/0	0.0000	21/4	0.0541	13/4	0.0880
rpoC1	2,031	–	–	9/3	0.0943	–	–
rpoC2	4,152	8/6	0.1570	–	–	18/19	0.2969
rps11	414	0/0	n.d.	3/2	0.2208	5/1	0.0651
rps12	369	0/0	n.d.	–	–	0/0	n.d.
rps14	300	0/0	n.d.	2/0	0.0000	2/0	0.0000
rps15	270	2/0	0.0000	4/2	0.1297	4/2	0.1302
rps16	252	0/0	n.d.	–	–	2/0	0.0000
rps18	303	0/0	n.d.	2/0	0.0000	4/0	0.0000
rps19	276	2/0	0.0000	6/1	0.0469	4/0	0.0000
rps2	708	1/0	0.0000	5/0	0.0000	3/1	0.0931
rps3	654	4/0	0.0000	6/1	0.0440	3/1	0.0895
rps4	603	1/0	0.0000	5/2	0.1154	2/1	0.1462
rps7	465	0/0	n.d.	1/1	0.3079	1/0	0.0000
rps8	402	0/0	n.d.	3/0	0.0000	1/0	0.0000
ycf1	5,709	–	–	–	–	–	–
ycf2	6,831	0/2	n.d.	10/11	0.2886	3/7	0.6136
ycf3	504	–	–	3/0	0.0000	5/0	0.0000
ycf4	552	0/0	n.d.	2/3	0.4184	3/1	0.0920

S/N, numbers of synonymous/non-synonymous substitutions. Ka/Ks estimated by the Nei–Gojobori method; n.d., not determined because Ks = 0. '–' indicates the gene was absent or of unequal length in the compared genome and was therefore excluded; infA, petN, psbC and ycf1 differed in length from all three congeners and are shown as '–' throughout. Concatenated-alignment values: R. *takesimensis* 0.068 (57,513 bp), R. *coreanus* 0.146 (51,123 bp), R. *chingii* 0.160 (57,789 bp). No individual gene exceeded Ka/Ks = 1 in any comparison.

Table S6. Candidate SNP panel for cultivar identification in *R. crataegifolius*, derived from nuclear resequencing data.

Marker	Position	Alleles	DAHE	DAHO	DB	DS	GDC	GDM	GDW	HGB	Min. depth
RcNu-01	OZ232402.1:27,407,814	A/G	BB	BB	AA	AA	AA	BB	AA	BB	11×
RcNu-02	OZ232400.1:932,131	G/T	BB	BB	BB	AA	BB	AA	BB	AA	14×
RcNu-03	OZ232401.1:5,003,032	G/A	BB	BB	AA	BB	AA	AA	BB	BB	16×
RcNu-04	OZ232403.1:12,029,618	A/G	AB	BB	BB	BB	BB	BB	BB	BB	17×
RcNu-05	OZ232399.1:1,175,884	A/G	BB	AB	BB	BB	AB	BB	AB	BB	14×

Genotypes are given as AA (homozygous reference), AB (heterozygous) and BB (homozygous alternate) with respect to the *Rubus idaeus* reference assembly GCA_965153425.1. Min. depth is the lowest per-sample read depth observed across the 121 bp window centred on each SNP. Within that window no position other than the target SNP differed among the eight cultivars, so flanking primers can be designed on conserved sequence. The five-marker genotype combination is unique for each cultivar: 'Dongakheuk' BB BB BB AB BB; 'Dongakhong' BB BB BB BB AB; 'Dabok' AA BB AA BB BB; 'Daesung' AA AA BB BB BB; 'Geumdongcheon' AA BB AA BB AB; 'Goldmoon' BB AA AA BB BB; 'Geumdongwang' AA BB BB BB AB; 'Hwangeumboll' BB AA BB BB BB.

Flanking sequences (60 bp on each side, target SNP in brackets):

```
RcNu-01  5'-
TCATTTCAGATAAAATTCTTCTTAATGCATTTTGGGTTCATCAGTACCCATCAATCACAT[A/G]AATTACGTGTTTCTTATCACACA
CTATATCCTTTCCTTTATCATTGATTATGAAAAGTTT-3'

RcNu-02  5'-
ACTAAGAGATATTTCATTTGGTGGTACGCTTGTATCAAGGCCAGTTAGATCTTTTAGATA[G/T]AGCTTATGTCATTATCTTTTCAG
TCTGTAGTTGGATTTGGTTCTAGACTCCTTGACATTT-3'

RcNu-03  5'-
CTTCTAATCCTGCCTGTTATTAAGGGACATGAAACTAACCCAACAACTATATTCCAATAT[G/A]AAATGCAGCAACTCTATAAACTC
CCCCACCTATGAGTCATTGGGTCATGGAAACTAATAT-3'

RcNu-04  5'-
AACCATCTTGGACAGTGAAACAGACCCTTCATCAGAATCAGTGGAAACACTTATATTTCC[A/G]CCAAGTTCTATATTTCTTTCGCC
TTCAGAAATAGTCCCCTGGGTATCAAAAAATTTTGCA-3'

RcNu-05  5'-
GAGGTTCTCAAGGTCAGGGATTCCAAATGATGTACTGCTGTTTCCAGTCACCCCGAACCC[A/G]TTGTTAGCACAGTCAGTGTTAGA
TATGGGATTCAGAGCTGGACCATTTAGCGGAGTGGAA-3'
```

Table S7. Repeat content of the eight *R. crataegifolius* cultivars (listed by cultivar name; accession PZ143672–PZ143679) and twelve published *Rubus* plastomes analyzed with identical parameters.

Taxon / cultivar	Accession	Genome (bp)	SSR	Comp.	Mono	Di	Tri	Tetra	Penta	Hexa	TRF	Disp.	F	P	R	C
'Dongakheuk'	this study	155664	69	8	41	13	4	10	1	0	44	22	10	9	3	0
'Dongakhong'	this study	155664	69	8	41	13	4	10	1	0	44	22	10	9	3	0
'Dabok'	this study	155713	82	14	53	13	5	11	0	0	43	17	9	8	0	0
'Daesung'	this study	155770	81	13	52	14	4	11	0	0	42	18	10	8	0	0
'Geumdongcheon'	this study	155713	82	14	53	13	5	11	0	0	43	17	9	8	0	0
'Goldmoon'	this study	155702	82	13	54	12	5	11	0	0	41	18	9	9	0	0
'Geumdongwang'	this study	155664	69	8	41	13	4	10	1	0	44	22	10	9	3	0
'Hwangeumboll'	this study	155703	74	9	46	13	5	9	1	0	45	23	10	9	4	0
R. crataegifolius	NC_039704	155714	83	14	54	13	5	11	0	0	43	17	9	8	0	0
R. takesimensis	NC_037991	155760	77	12	48	14	4	11	0	0	42	20	10	10	0	0
R. trifidus	NC_046585	155823	71	11	46	14	3	7	0	1	46	19	10	8	1	0
R. chingii	NC_050296	155843	79	14	56	13	4	5	1	0	50	29	12	17	0	0
R. corchorifolius	NC_056942	155526	80	16	58	13	4	5	0	0	57	23	11	11	1	0
R. hirsutus	OK127882	156020	76	8	53	10	2	10	1	0	53	17	9	8	0	0
R. sumatranus	PP737792	155935	77	12	53	12	4	8	0	0	51	22	10	9	3	0
R. coreanus	NC_042715	155785	66	12	48	7	3	6	2	0	66	20	10	10	0	0
R. phoenicolasius	NC_057144	155144	68	9	48	10	1	9	0	0	38	18	7	11	0	0
R. parvifolius	NC_060617	155906	62	9	44	7	3	7	1	0	68	28	14	12	1	1
R. arcticus	NC_062417	156668	69	11	41	15	4	9	0	0	53	21	10	10	1	0
R. buergeri	NC_072261	156276	63	7	46	8	2	7	0	0	54	20	8	11	1	0

All 20 plastomes were analyzed with the same settings as in Materials and Methods: MISA minimum repeat numbers 1-10, 2-5, 3-4, 4-3, 5-3, 6-3; Tandem Repeats Finder 2 7 7 80 10 50 500; dispersed repeats by BLASTn self-alignment, ≥ 30 bp, Hamming distance ≤ 3, with the IR-derived palindromic repeat excluded. SSR, total SSRs; Comp., SSRs in compound formation; Mono–Hexa, SSRs by repeat-unit size; TRF, tandem repeats (Tandem Repeats Finder); Disp., dispersed repeats in total and by type (F forward, P palindromic, R reverse, C complement). Cultivar values are those of Table 2.

Table S8. Length polymorphism of plastid SSR loci among the eight *R. crataegifolius* cultivars.

Locus	Position	Type	Motif ('Dongakhong')	DAHE	DAHO	DB	DS	GDC	GDM	GDW	HGB	NC_039704	NC_037991
cpSSR-001	214	p1	(A)11	10	10	28	22	28	28	10	10	28	29
cpSSR-004	4335	p1	(A)11	9	9	10	11	10	10	9	9	10	11
cpSSR-005	4502	p1	(T)10	10	10	10	10	10	10	10	11	10	9
cpSSR-006	6138	c/p1	(C)12	68	68	67	69	67	67	68	71	67	68
cpSSR-009	7220	p1	(A)11	5	5	11	12	11	11	5	5	11	12
cpSSR-010	8750	p1	(A)14	14	14	12	13	12	12	14	14	12	14
cpSSR-013	14007	c/p1	(T)10	22	22	22	22	22	22	22	21	22	21
cpSSR-017	28357	p1	(T)10	11	11	10	11	10	10	11	11	10	10
cpSSR-018	29264	c	(G)10cac(TA)6atatgtaa ttga…	52	52	64	64	64	64	52	52	64	63
cpSSR-019	31709	p1	(T)11	8	8	9	11	9	9	8	8	9	11
cpSSR-020	32356	p1	(T)10	11	11	11	11	11	11	11	10	11	11
cpSSR-021	32834	p1	(A)11	10	10	11	10	11	11	10	10	11	11
cpSSR-024	43433	p1	(T)10	9	9	9	9	9	9	9	10	9	9
cpSSR-026	47028	p2	(TA)5	10	10	9	10	9	9	10	10	9	10
cpSSR-027	47279	p1	(A)11	11	11	27	28	27	27	11	16	27	28
cpSSR-028	47536	p5	(CATTT)3	15	15	10	10	10	10	15	15	10	10
cpSSR-031	47993	p1	(A)10	9	9	10	10	10	10	9	9	10	9
cpSSR-033	50228	c/p2	(AT)5	54	54	56	57	56	56	54	54	56	56
cpSSR-035	52522	c/p2	(TA)5ttatatacat(TA)8	36	36	36	36	36	23	36	36	36	36
cpSSR-037	60576	p1	(T)12	12	12	12	10	12	12	12	11	12	12
cpSSR-039	61667	p1	(T)10	10	10	10	10	10	10	10	11	10	11
cpSSR-041	63939	p1	(T)10	10	10	13	13	13	13	10	11	13	13
cpSSR-042	64165	p1	(G)12	11	11	13	13	13	13	11	12	13	11
cpSSR-043	64668	p1	(T)10	10	10	10	10	10	10	10	11	10	10
cpSSR-044	67763	p1	(T)10	9	9	9	10	9	9	9	9	9	10

Locus	Position	Type	Motif ('Dongakhong')	DAHE	DAHO	DB	DS	GDC	GDM	GDW	HGB	NC_039704	NC_037991
cpSSR-046	69255	c	(T)11cgaaaaataggaaatc caga…	72	72	72	73	72	72	72	73	72	72
cpSSR-047	70476	p1	(T)12	12	12	11	12	11	11	12	13	11	12
cpSSR-048	71467	p1	(T)14	14	14	11	11	11	11	14	11	11	11
cpSSR-049	72481	c/p1	(T)10	19	19	21	18	21	22	19	20	21	21
cpSSR-051	78948	p1	(A)14	14	14	10	13	10	10	14	14	10	12
cpSSR-052	81045	p1	(T)10	9	9	9	9	9	9	9	10	9	9
cpSSR-053	81648	p1	(T)12	12	12	13	14	13	13	12	11	13	12
cpSSR-054	82155	p1	(T)12	9	9	11	12	11	11	9	11	11	12
cpSSR-055	82954	c/p1	(T)10attattctatcgaattga ct…	120	120	121	121	121	121	120	120	121	120
cpSSR-056	83442	p1	(T)10	8	8	9	9	9	10	8	8	10	9
cpSSR-058	85028	c	(TTA)4(T)12	25	25	25	24	25	25	25	25	25	22
cpSSR-062	114698	p4	(ATTT)3	18	18	14	14	14	14	18	18	14	14
cpSSR-063	114842	p1	(A)11	10	10	11	11	11	11	10	10	11	11
cpSSR-064	115229	p3	(TAA)4	6	6	6	6	6	6	6	12	6	6
cpSSR-067	121771	p1	(A)11	10	10	12	13	12	12	10	11	12	12
cpSSR-068	122018	p1	(T)13	15	15	14	13	14	14	15	15	14	13
cpSSR-069	122643	c	(A)12tgaagatatctcctcta ctc…	64	64	65	65	65	65	64	65	65	65
cpSSR-070	124873	p1	(T)10	9	9	10	9	10	10	9	10	10	9

Of the 76 SSR loci called by MISA in at least one cultivar (loci overlapping in the alignment merged), the 43 whose ungapped length differs among the eight cultivars are listed; values are the ungapped length (bp) of the locus in each sequence, read through the MAFFT alignment. Position is the start coordinate in 'Dongakhong' (PZ143673). Type: p1–p5, perfect mono- to pentanucleotide repeat; c, compound. Motif is the repeat as called in 'Dongakhong' (truncated where long). The combined length profiles define five genotypes that coincide with the five plastome haplotypes: 'Dongakheuk' = 'Dongakhong' = 'Geumdongwang' (group A) and 'Dabok' = 'Geumdongcheon' (group B) are identical at every locus. The published *R. crataegifolius* plastome NC_039704 and *R. takesimensis* NC_037991 are shown for comparison. Cultivar abbreviations as in Table S6.

Table S9. Reference-free estimates of heterozygosity and genome size for the eight *R. crataegifolius* cultivars from 21-mer spectra (GenomeScope 2.0).

Cultivar	Heterozygosity (%)	Haploid genome length (Mb)	Repeat length (Mb)	k-mer coverage (het. peak)	Model fit (%)	Read error rate (%)
Dongakheuk	1.70–1.74	260.6	106.9	10.4	81.9–99.3	0.09
Dongakhong	1.66–1.72	258.0	104.8	10	81.5–99.2	0.12
Dabok	1.66–1.71	237.7	84.4	10.1	82.2–99.2	0.12
Daesung	1.81–1.84	239.9	88.5	11.8	81.6–99.2	0.11
Geumdongcheon	1.76–1.79	244.4	91.4	11.5	82.4–99.3	0.12
Goldmoon	1.48–1.52	239.3	86.3	10.8	81.8–99.3	0.13
Geumdongwang	1.66–1.69	239.8	86.9	11.9	82.4–99.2	0.11
Hwangeumboll	1.71–1.74	244.1	91.1	10.9	82.3–99.2	0.11

21-mers were counted from the quality-trimmed read pairs of each cultivar with Jellyfish v2.3.1 (canonical k-mers) and the histogram fitted with GenomeScope 2.0 under a diploid model (k = 21, k-mers with coverage > 2,000 excluded). Heterozygosity is given as the minimum–maximum of the model fit; genome and repeat lengths are the means of the fitted minimum and maximum. For comparison, the same method gave 1.54% for *R. idaeus* 'Autumn Bliss', 0.45% for 'Malling Jewel' and 0.80% for *R. chingii* in the respective genome papers (main-text references 39 and 40).

Note S1. Selection and properties of the candidate SNP panel.

Of the SNPs passing the filters described in Materials and Methods, 400 were retained for panel construction. A minimal combination was assembled greedily, choosing at each step the genotype pattern that separated the

largest number of the 28 cultivar pairs. Three homozygous-differentiating SNPs separated 26 pairs; the two most similar pairs, 'Dongakheuk'–'Dongakhong' and 'Dabok'–'Geumdongcheon', required two further SNPs at which one cultivar is heterozygous. Each marker was scored at 11–17× minimum depth in all cultivars, and alternate-allele fractions at the target sites were 0% or 100% for every homozygous call. Within the 121 bp window surrounding each marker no position other than the target SNP differed among the cultivars, so flanking sequence is conserved and available for assay design (Table S6). Two limitations should be borne in mind. First, the markers were selected on the same eight cultivars that they are then shown to separate; with 4,872,213 cultivar-polymorphic SNPs available, such a combination is expected to exist and the demonstration is therefore internal. Second, redundancy is limited, as only one to three alternative SNPs sharing the same genotype pattern passed the same filters, so failure of a single assay would leave some cultivar pairs unresolved. Conversion into KASP or CAPS assays and validation on independent plant material remain to be done. Minimal SNP sets read as a binary barcode are an established route to cultivar identification: for Korean perilla, minimal sets of five and six markers were derived from 150 KASP markers validated across 48 cultivars [49]. All five markers lie within the strict SNP set defined in the revised Materials and Methods (MQ ≥ 50, per-sample depth 8–40×, allele balance 0.2–0.8); the heterozygous calls on which RcNu-04 and RcNu-05 depend have alternate-allele fractions of 0.50–0.60 at 20–26× depth, and every homozygous call is free of minor-allele reads. RcNu-04 replaces a marker of identical genotype pattern used in the original submission, whose heterozygous call rested on 3 of 25 reads.

Reference [49], cited above, as numbered in the main text: Kim, J.I.; Lee, M.H.; Kim, S.W.; Lee, E.; Kim, S.; Lee, J.; Lee, H.; Kim, K.Y.; Oh, E.; Kim, M.Y.; et al. Development and application of SNP markers to discriminate Korean Perilla (*Perilla frutescens*) varieties using genomic sequence variations. *Sci. Rep.* 2025, *16*, 3097.

Note S2. Why the 'Daesung'–*R. takesimensis* relationship could not be tested with biparentally inherited markers.

No nuclear sequence data suitable for this purpose are publicly available for *R. takesimensis*. In GenBank the species is represented by two plastomes, chloroplast intergenic markers from Ulleung Island populations, two nrDNA ITS clones and a single LEAFY intron 2 sequence, with no genome assembly and no raw sequencing reads (NCBI, accessed August 2026). Neither of the two nuclear markers proved usable. First, in a read-level analysis of the ITS region in our own cultivars, most variable positions were polymorphic within individuals, with alternate-allele fractions of 16–79% at 300–600× depth, as expected for a multi-copy rDNA array; consensus ITS sequences are therefore not a reliable basis for comparison at this level of divergence. Second, the published *Rubus* LEAFY intron 2 sequences align over their full length at 99.6% identity to the sequence annotated as the mitochondrial genome in the *R. idaeus* reference assembly, whereas the best match on a chromosome covers only a third of the fragment at 92.7% identity; in our cultivars the region is recovered at organellar rather than nuclear read depth (300–500× against ~21× genome-wide). We did not assemble the *R. crataegifolius* mitochondrion and so cannot establish the location of this copy directly, but on this evidence the fragment is unlikely to provide a biparentally inherited signal.

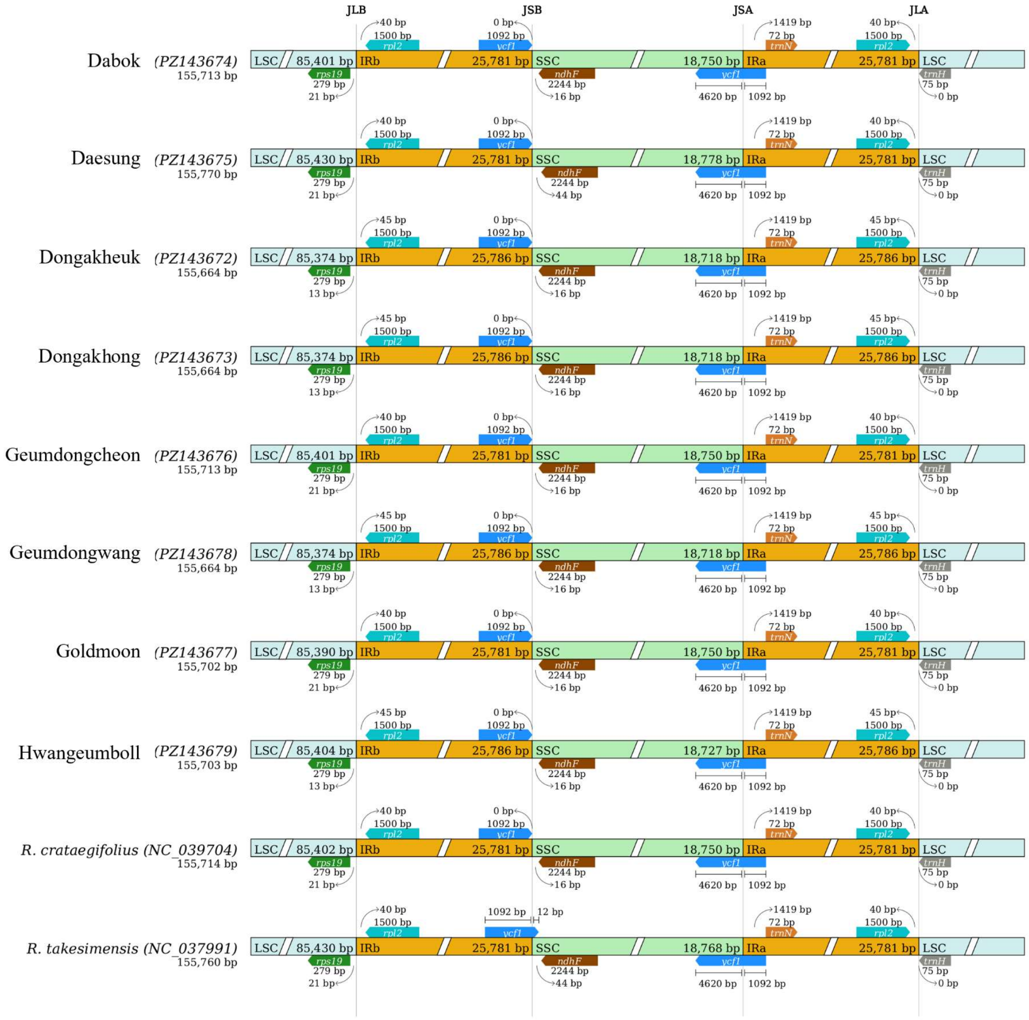

Figure S1. Comparison of inverted repeat (IR) region boundaries among eight *R. crataegifolius* cultivars and two reference sequences (*R. crataegifolius* NC_039704; *R. takesimensis* NC_037991). Four junctions are shown: JLB (LSC/IRb), JSB (IRb/SSC), JSA (SSC/IRa), and JLA (IRa/LSC). Colored boxes represent genes, with distances (in bp) from the nearest junction indicated. The 1,092 bp ycf1 box adjacent to JSB is the truncated pseudogene copy in the IRb.

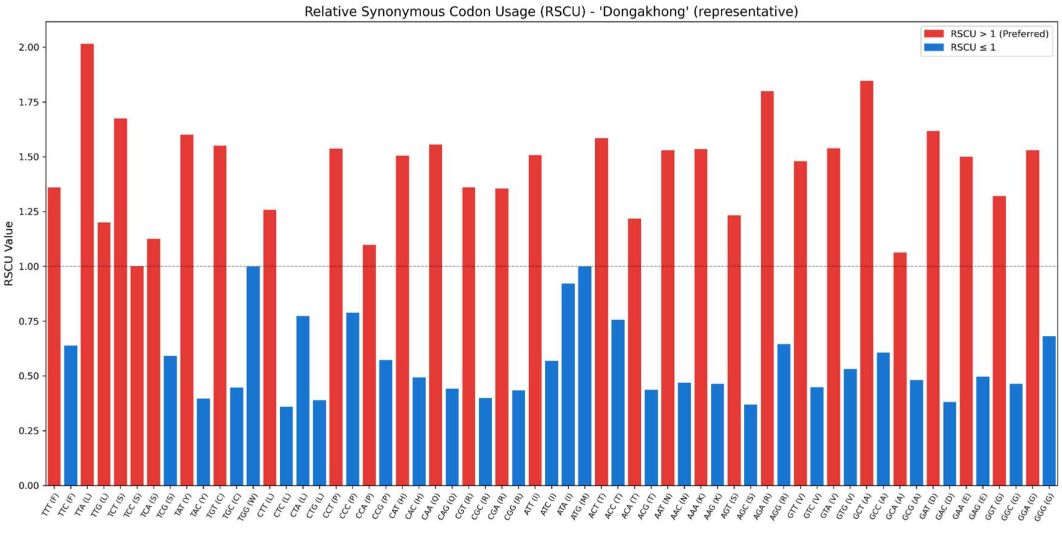

Figure S2. Relative synonymous codon usage (RSCU) values of 79 protein-coding genes in the *R. crataegifolius* 'Dongakhong' chloroplast genome (representative of all eight cultivars, which showed virtually identical RSCU profiles). Codons with RSCU > 1 indicate relative over-representation; those with RSCU < 1 indicate under-representation. Stop codons are excluded.

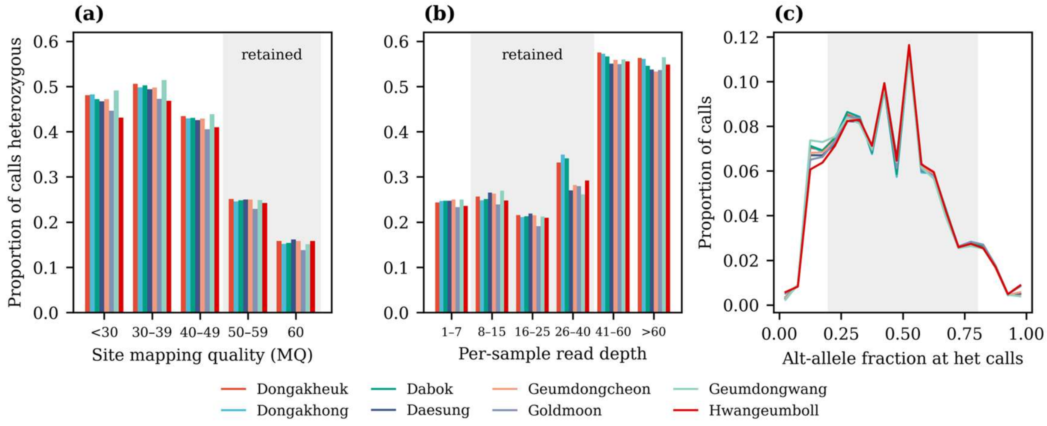

Figure S3. Quality assessment of nuclear heterozygous calls in the eight *R. crataegifolius* cultivars (8,308,205 biallelic SNPs before removal of fixed interspecific differences). (a) Proportion of genotype calls that are heterozygous, by site mapping quality (INFO/MQ). (b) The same by per-sample read depth. (c) Distribution of the alternate-allele fraction (FORMAT/AD) at heterozygous calls. Gray shading marks the ranges retained in the strict SNP set (MQ ≥ 50; depth 8–40×; allele balance 0.2–0.8). The excess of heterozygous calls at low mapping quality and at roughly twice the mean depth is the signature of paralogous regions of the *R. crataegifolius* genome collapsing onto single copies of the *R. idaeus* reference.

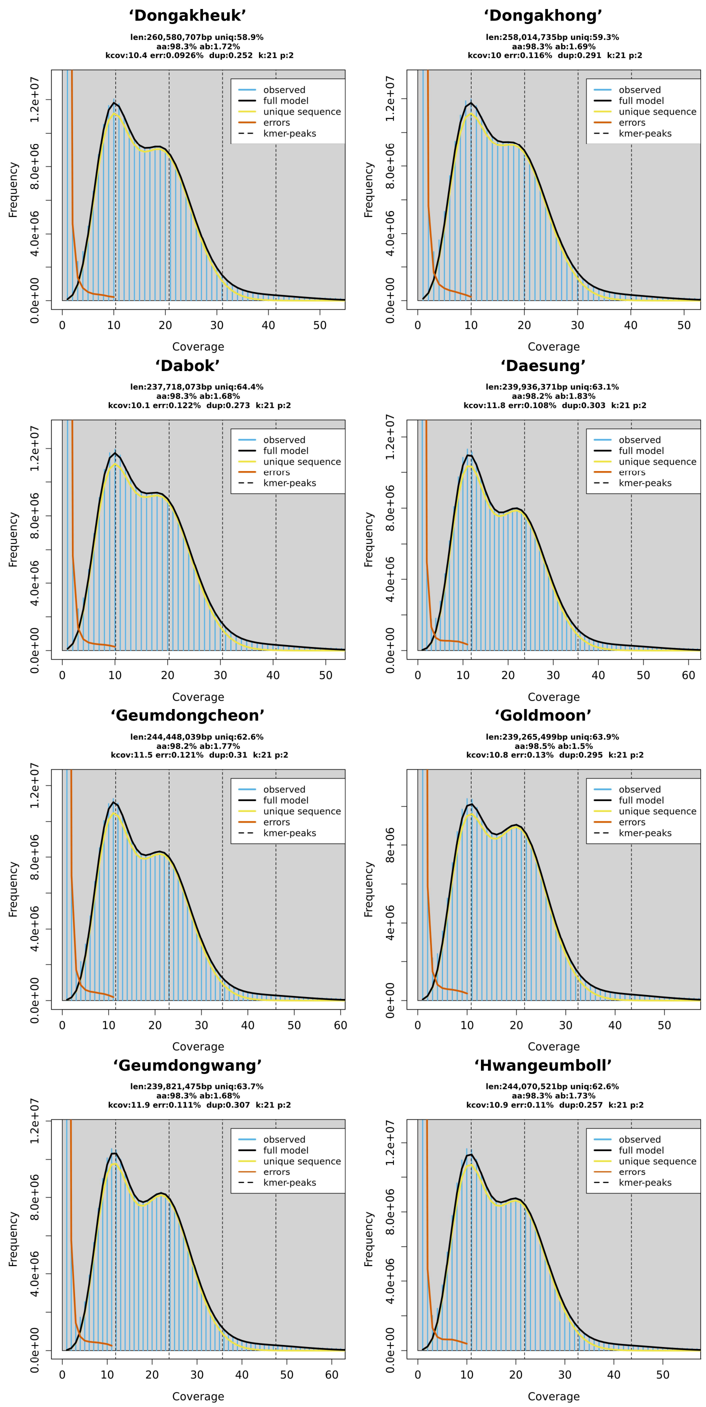

Figure S4. GenomeScope 2.0 fits to the 21-mer spectra of the eight *R. crataegifolius* cultivars (linear scale; cultivar name above each panel). Blue bars, observed 21-mer counts; black line, full model; yellow, unique sequence; orange, error k-mers; dashed lines, fitted k-mer peaks. The header of each panel gives the GenomeScope estimates of haploid length (len), unique fraction (uniq), homozygous and heterozygous k-mer proportions (aa, ab) and heterozygous k-mer coverage (kcov). Each spectrum shows the heterozygous peak at 10–12× and the homozygous peak at twice that coverage; the fitted parameters are tabulated in Table S9.
